# Transcriptional repression of Myc underlies the tumour suppressor function of AGO1 in *Drosophila*

**DOI:** 10.1242/dev.190231

**Published:** 2020-06-11

**Authors:** Olga Zaytseva, Naomi C. Mitchell, Linna Guo, Owen J. Marshall, Linda M. Parsons, Ross D. Hannan, David L. Levens, Leonie M. Quinn

**Affiliations:** 1 Department of Cancer Biology and Therapeutics, The John Curtin School of Medical Research, The Australian National University, Canberra, ACT 2600, Australia; 2 University of Tasmania, Hobart, Tasmania 7000, Australia; 3 Center for Cancer Research, National Cancer Institute, NIH, Bethesda, MD 20892, USA

**Keywords:** Argonaute, *Drosophila*, Myc, Proliferation, Psi, Transcription

## Abstract

Here, we report novel tumour suppressor activity for the *Drosophila* Argonaute family RNA-binding protein AGO1, a component of the miRNA-dependent RNA-induced silencing complex (RISC). The mechanism for growth inhibition does not, however, involve canonical roles as part of the RISC; rather, AGO1 controls cell and tissue growth by functioning as a direct transcriptional repressor of the master regulator of growth, Myc. AGO1 depletion in wing imaginal discs drives a significant increase in ribosome biogenesis, nucleolar expansion and cell growth in a manner dependent on Myc abundance. Moreover, increased *Myc* promoter activity and elevated *Myc* mRNA in AGO1-depleted animals requires RNA polymerase II transcription. Further support for transcriptional AGO1 functions is provided by physical interaction with the RNA polymerase II transcriptional machinery (chromatin remodelling factors and Mediator Complex), punctate nuclear localisation in euchromatic regions and overlap with Polycomb Group transcriptional silencing loci. Moreover, significant AGO1 enrichment is observed on the *Myc* promoter and AGO1 interacts with the *Myc* transcriptional activator Psi. Together, our data show that *Drosophila* AGO1 functions outside of the RISC to repress *Myc* transcription and inhibit developmental cell and tissue growth.

This article has an associated ‘The people behind the papers’ interview.

## Abstract (precis)

**Highlighted Article:** In the *Drosophila* wing, the Argonaute family protein AGO1 acts independently of the miRNA-silencing pathway to restrict tissue growth by directly repressing transcription of the master growth regulator Myc.

## INTRODUCTION

Tightly coordinated regulation of cell and tissue growth is essential for animal development. Decreased growth leads to small organs and diminished body size, whereas heightened proliferative growth is associated with genomic instability and cancer. The MYC transcription factor and growth regulator has been studied extensively since its identification as an oncogene in the early 1980s (Vennstrom et al., 1982), when MYC overexpression caused by chromosomal translocation was found to drive malignant transformation in Burkitt's lymphoma (Dalla-Favera et al., 1982; Taub et al., 1982). Research in subsequent decades implicated increased MYC in progression of most tumours (Dang, 2012; Liao and Dickson, 2000; Meyer and Penn, 2008). In normal adult tissues, MYC expression is relatively low and generally restricted to cells with regenerative and proliferative potential (Marcu et al., 1992). Even small increases in MYC abundance are sufficient to promote proliferative cell growth (reviewed by Dang, 2010; Levens, 2010; Zaytseva and Quinn, 2017); thus, understanding the molecular control of *MYC* expression can provide crucial insight into the mechanisms of MYC dysregulation in cancer.

In normal cells, MYC is regulated by signalling inputs from a diverse array of developmental and growth signalling pathways (Zaytseva and Quinn, 2017). The many cellular signalling inputs converging on *MYC* transcription are integrated by FUBP1, a KH domain protein that binds single-stranded DNA and interacts with the general transcription factor complex TFIIH to modulate *MYC* promoter output (Chung and Levens, 2005; Chung et al., 2006; He et al., 2000; Liu et al., 2006; Zhang and Chen, 2013). The mammalian FUBP family comprises three proteins (FUBP1-3) (Zhang and Chen, 2013), which are represented by one orthologue in *Drosophila*, Psi. Like FUBP1, Psi also interacts with the RNA polymerase II (RNA Pol II) transcriptional machinery, particularly the transcriptional Mediator (MED) complex, to pattern *Myc* transcription and cell and tissue growth in the *Drosophila* wing epithelium (Guo et al., 2016). In addition to roles in transcription, Psi binds RNA via the KH domains and interacts with the spliceosome to regulate mRNA splicing (Labourier et al., 2001; Wang et al., 2016). Although co-immunoprecipitation (co-IP) mass spectrometry detected Psi in complex with the Argonaute protein AGO1 (Guo et al., 2016), the potential significance of this interaction is unknown.

Argonaute proteins comprise the core of the RNA-induced silencing complex (RISC), which uses noncoding RNA as a guide to target mRNAs for post-transcriptional gene silencing. *Drosophila* AGO2 is best characterised as part of the siRNA-induced silencing complex (siRISC) (Okamura et al., 2004), whereas AGO1 predominantly functions in microRNA-induced silencing complexes (miRISCs) and post-transcriptional mRNA silencing (Förstemann et al., 2007). Of importance to this study, AGO1-mediated mRNA silencing has been implicated in transcript destabilisation and translational repression of *Myc* in flies (Daneshvar et al., 2013) and humans (Challagundla et al., 2011). Here, we report a novel role for AGO1 as a direct *Myc* transcriptional repressor and demonstrate that this underlies cell growth inhibition. AGO1 depletion not only increases *Myc* promoter activity, mRNA and protein abundance, but the elevated *Myc* expression requires RNA Pol II transcriptional activity. Localisation to the nucleus, together with interaction with transcriptional machinery and significant AGO1 enrichment on the *Myc* promoter suggests, in addition to the established roles in miRNA silencing in the cytoplasm, AGO1 constrains *Myc* transcription to control cell and tissue growth during *Drosophila* development.

## RESULTS

### AGO1 interacts with Psi and RNA Pol II transcriptional machinery

**Fig. 1.**
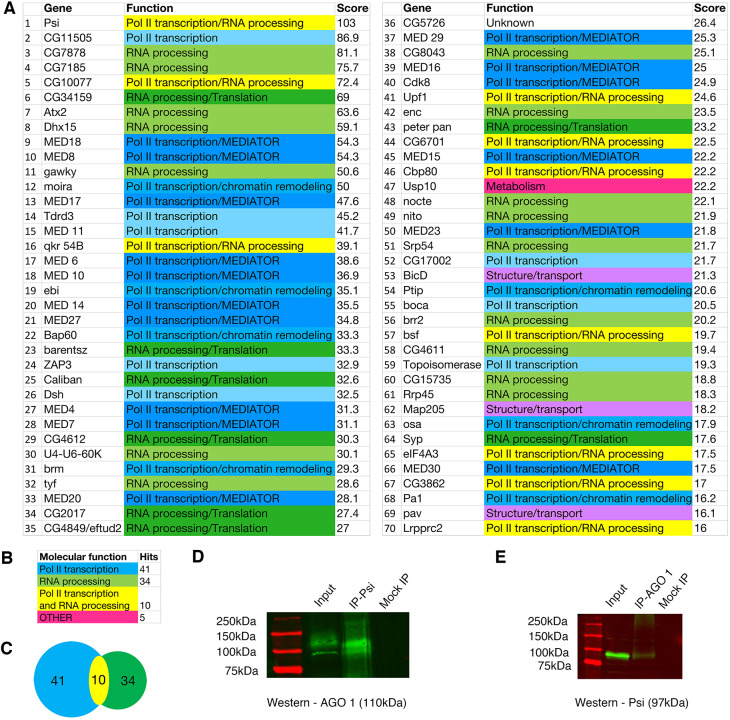
**AGO1 interacts with RNA Pol II transcriptional machinery and RNA processing factors.** (A) List of top 70 AGO1 interactors from the *Drosophila* Protein Interaction Map (DPiM) dataset (Guruharsha et al., 2011). (B) Summary of ontology classes for the top 70 AGO1 interactors. (C) Intersection of interactors with functions in RNA Pol II transcription and/or RNA processing. (D,E) Co-IP of endogenous Psi and AGO1 from wild-type third instar larvae. (D) IP with anti-Psi and western blot with anti-AGO1 (110 kDa). (E) IP with anti-AGO1 and western blot with anti-Psi (97 kDa). Mock IP refers to no-antibody control.

The single-stranded DNA/RNA binding protein Psi has essential roles in *Myc* transcriptional control and RNA processing in *Drosophila*. In addition to physically and genetically interacting with the RNA Pol II transcriptional machinery, the *Drosophila* Protein Interaction Map (DPiM) large scale co-IP mass spectrometry (Guruharsha et al., 2011) suggested association between Psi and AGO1 (Guo et al., 2016). Our analysis of the DPiM identified Psi as the most frequent AGO1 interacting partner (Fig. 1A). Ontological class analysis for the top 70 AGO1 interactors revealed RNA processing factors (49%), as expected; however, most (59%) interactors had ascribed functions in RNA Pol II transcription (Fig. 1A-C, note 10 factors are implicated in both transcription and RNA processing). As the DPiM studies were performed *in vitro* with overexpressed tagged proteins in *Drosophila* S2 cell lines, we validated the interaction between endogenous AGO1 and Psi *in vivo* using co-IP from wild-type third instar larval imaginal tissue. Immunoprecipitation using anti-Psi antibody, followed by anti-AGO1 western blot detected a 110 kDa band for AGO1 (Fig. 1D), whereas reciprocal IP with anti-AGO1 antibody precipitated the 97 kDa Psi band (Fig. 1E). The observation that endogenous AGO1 and Psi form a complex *in vivo* led us to investigate potential genetic interactions between AGO1 and Psi.

### Psi-dependent growth is sensitive to AGO1 abundance

**Fig. 2.**
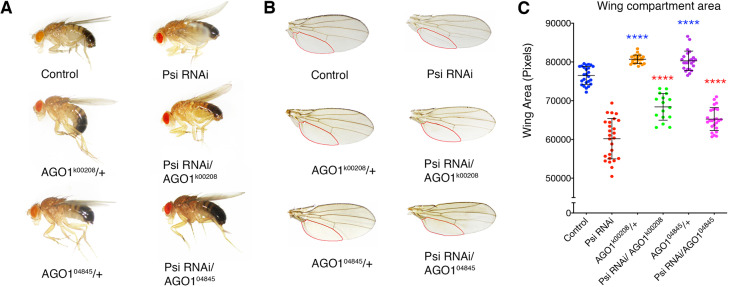
**Impaired cell and tissue growth in the Psi knockdown wing is sensitive to AGO1 levels.** (A,B) Adult flies (A) and wings (B), with compartment below vein L5 outlined in red (genotypes in the *ser-GAL4* background as marked). (C) Quantification of compartment area below vein L5 in individual adult wings in the *ser-GAL4* background. Data points are measurements per individual wing, with mean±s.d. shown. Both *AGO1* mutants were compared with the control; *AGO1* mutants in *Psi* RNAi background were compared with Psi knockdown alone. Unpaired two-tailed *t*-test was used for all comparisons. ****P<0.0001.

Psi knockdown in the dorsal wing compartment results in a ‘wings up’ phenotype as impaired cell and tissue growth of the top layer of the wing results in torsional strain and wing bending (Guo et al., 2016). We therefore tested whether this was modulated by AGO1 depletion, using two independent P-element insertion mutants (*AGO1^k00208^* and *AGO1^04845^*). Interestingly, *AGO1* heterozygosity alone was sufficient to increase wing size, suggesting that AGO1 normally constrains growth. Moreover, heterozygosity for either *AGO1* mutant suppressed impaired tissue growth caused by Psi depletion (Fig. 2A-C). Thus, AGO1 normally functions as a negative growth regulator and the wing overgrowth associated with AGO1 reduction is dependent upon Psi. However, we need to be somewhat cautious in our interpretation of these data as either genotype alone results in a phenotype, where the outcome may be an intermediate phenotype between the larger wings from AGO1 heterozygotes and impaired growth from Psi knockdown.

### AGO1 depletion drives cell growth in a Myc-dependent manner

To further examine the cellular basis of the tissue overgrowth associated with AGO1 depletion in specific compartments of the larval wing, we used two independent non-overlapping *AGO1* RNAi lines. We first demonstrated efficient mRNA knockdown for both *AGO1* lines in the wing (Fig. S1A) and dorsal compartment-specific protein knockdown 24 h after induction of *ser-*GAL4 (Fig. S1B). As pupal lethality and dorsal compartment cell death were associated with constitutive *ser-*GAL4 driven AGO1 knockdown (Fig. S2), the baculoviral caspase inhibitor p35 was co-expressed to prevent apoptosis (Hay et al., 1994) and enable investigation of potential changes to cell growth.

**Fig. 3.**
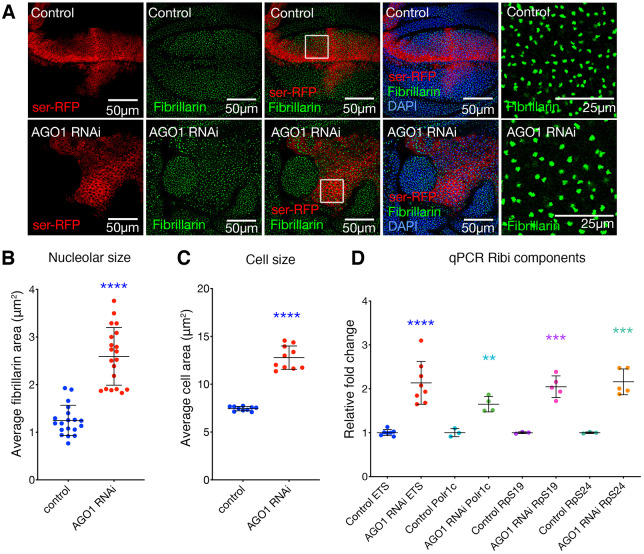
**AGO1 knockdown increases ribosome biogenesis.** (A) Third instar wing discs with *ser*-GAL4-driven *AGO1* RNAi in the *UAS-p35* background compared with control (*ser*-GAL4, *p35* alone), marked with *UAS-RFP* and stained with anti-fibrillarin (green) and DAPI (blue). Far right panel shows magnification of the boxed areas. (B) Quantification of average nucleolar area of approximately 50-100 nucleoli within the dorsal compartment, taken from a confocal *z*-section through the wing discs with *ser*-GAL4 expression in *p35* background (data points are average measurements per image, with mean±s.d. shown). *AGO1* RNAi was compared with control. (C) Quantification of cell area marked by CD8-RFP, calculated as average of approximately 50-100 cells per wing disc, in the dorsal compartment of wing *ser*-GAL4 in *p35* background (data points are average measurements per image, with mean±s.d. shown). *AGO1* RNAi was compared with control. (D) qPCR for 47S pre-RNA (ETS), RNA Pol I subunit (*Polr1c*) and ribosomal proteins (*RpS19* and *RpS24*) in wing discs (20 discs pooled for each data point, mean±s.d. shown). Expression was performed for 2 days using *tub*-GAL4 in the background of *tub*-GAL80ts. AGO1 knockdown in the wing significantly increased abundance of *Polr1c*, 47S rRNA 5′-ETS, *RpS19* and *RpS24* (*P*=0.0004 and *P*=0.0006, respectively) compared with the corresponding control. Unpaired two-tailed *t*-test was used for all comparisons. **P<0.01, ***P<0.001, ****P<0.0001.

Cell growth requires ribosomal RNA (rRNA) synthesis, processing and assembly with ribosomal proteins (RPs) into 40S and 60S ribosomal subunits in the nucleolus. Thus, the size of this structure, as measured by nucleolar-specific fibrillarin antibody, provides an indirect measure of ribosome biogenesis (Mitchell et al., 2015). AGO1 depletion significantly increased nucleolar size (Fig. 3A,B), and cell size (Fig. 3C), suggesting that AGO1 normally functions to inhibit cell growth. Consistent with AGO1 depletion driving nucleolar expansion, at least in part a result of increased rDNA transcription, transient *AGO1* knockdown significantly increased pre-rRNA levels and *Polr1c* (RNA polymerase I (subunit) mRNA (Fig. 3D). AGO1 depletion also significantly increased levels of the ribosomal protein subunits *RpS19* and *RpS24* (Fig. 3D). Together, these data suggest that AGO1 normally inhibits ribosome biogenesis and cell growth in the wing imaginal disc. Chromatin immunoprecipitation (ChIP) sequencing studies have previously identified AGO2 binding throughout the 47S region of the consensus rRNA gene in human cell lines (Atwood et al., 2016), suggesting direct roles for AGO proteins in rDNA transcription and/or rRNA processing. However, these observations do not explain the increase in expression of RNA Pol II-transcribed ribosomal proteins and RNA Pol I subunits, nor the increase in overall cell growth, which requires coordinated activity from all three RNA polymerases, RNA Pol I, II and III (the last required for transcription of 5S rRNA).

**Fig. 4.**
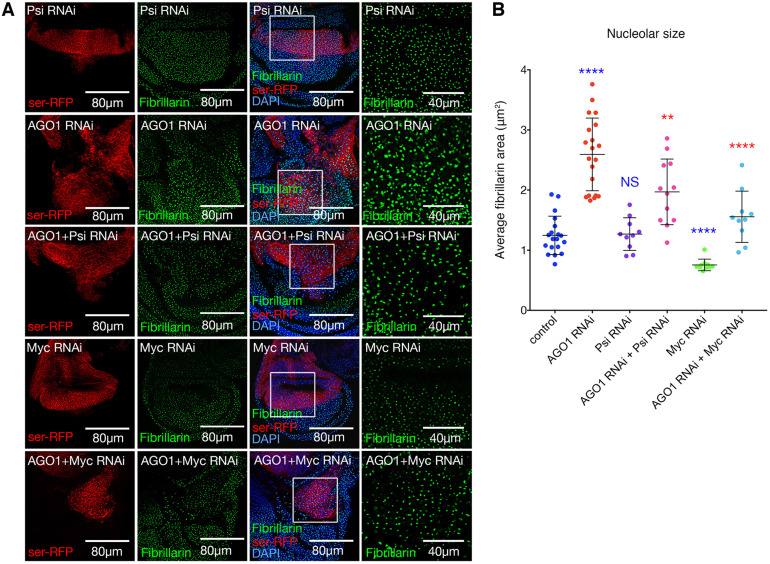
**Increased nucleolar size as a result of AGO1 knockdown is dependent on Psi and Myc.** (A) *ser*-GAL4-driven RNAi in RFP-labelled cells for the indicated genotypes in wing discs stained with anti-fibrillarin (green) and DAPI (blue). Far right panel shows magnification of regions in the white squares. (B) Quantification of average nucleolar area of approximately 50-100 nucleoli within the dorsal compartment, taken from a confocal *z*-section through the wing discs with *ser*-GAL4 expression in *p35* background (data points are average measurements per image, mean±s.d. shown). *Myc* RNAi reduced nucleolar size compared with control. Psi or Myc co-depletion significantly reduced nucleolar size in the *AGO1* RNAi background compared with AGO1 knockdown alone (*P*<0.0001 and *P*=0.0065 for Myc and Psi, respectively). Unpaired two-tailed t-test was used for all comparisons. NS, not significant; **P<0.01, ****P<0.0001.

MYC drives cell growth by stimulating transcription of all three RNA polymerases to upregulate ribosome production (Poortinga et al., 2014). MYC directly stimulates the initiation of RNA Pol I-mediated transcription in mammals (Arabi et al., 2005; Grandori et al., 2005; Shiue et al., 2009) and activates transcription of RNA Pol II-transcribed genes encoding the ribosomal proteins (RPs), rRNA processing factors and components of the nucleolus essential for ribosome biogenesis (Grandori et al., 2005; Grewal et al., 2005; Poortinga et al., 2011). Furthermore, MYC directly activates RNA Pol III transcription to increase 5S rRNA expression (for assembly of the large 60S ribosomal subunit) and tRNA for translation of mRNA into protein (Fernandez et al., 2003; Gomez-Roman et al., 2006; Oskarsson and Trumpp, 2005). In *Drosophila*, Myc depletion reduced nucleolar expansion in AGO1 knockdown wing cells to the control range (Fig. 4A,B). Co-depletion of the Myc-regulator Psi also significantly decreased nucleolar size compared with AGO1 knockdown alone (Fig. 4A,B). Importantly, given the intermediate effect between *ser*-GAL4 driven Psi knockdown and AGO1 heterozygotes in the adult wing (Fig. 2), Psi depletion alone did not modify nucleolar size. Thus, the increased ribosome biogenesis and cell overgrowth associated with AGO1 depletion is dependent on both Psi and Myc.

### AGO1 depletion increases Myc abundance and function

**Fig. 5.**
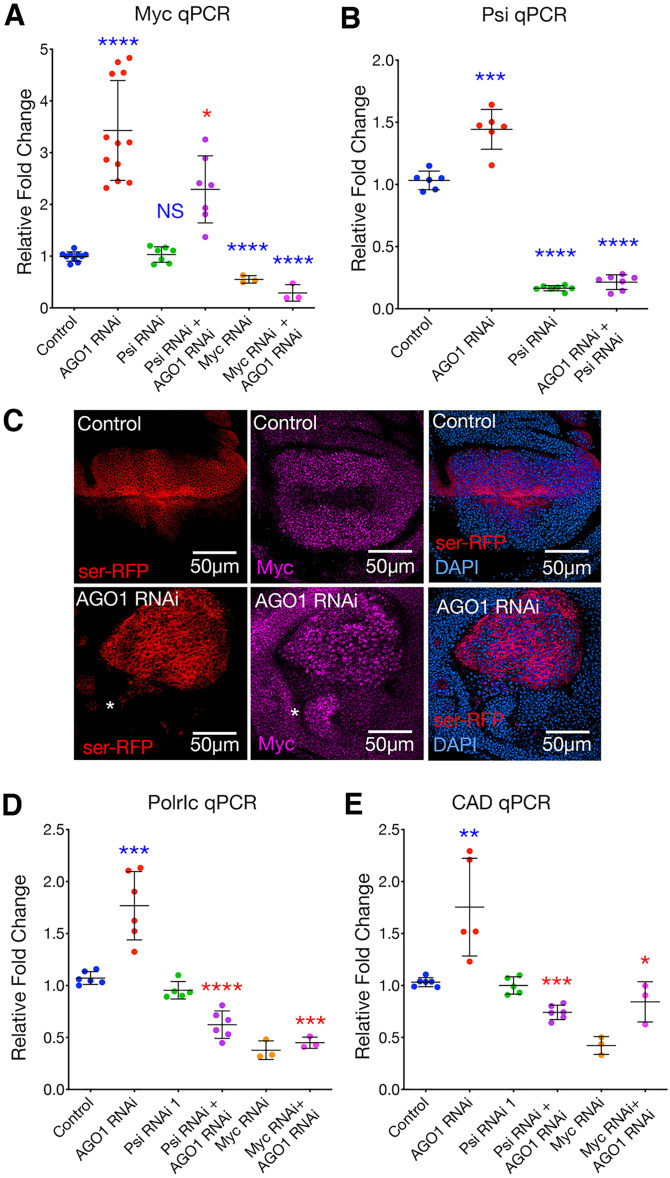
**AGO1 knockdown increases Myc abundance and function in larval wing discs.** (A) *Myc* qPCR following *AGO1* RNAi and/or *Psi* RNAi knockdown (20 discs pooled for each data point, mean±s.d. shown). Expression was performed for 2 days using *tub*-GAL4 in the background of *tub*-GAL80ts. AGO1 knockdown significantly increased *Myc* mRNA, compared with control. Psi co-knockdown significantly reduced *Myc* mRNA compared with *AGO1* RNAi alone (*P*=0.0134). *Myc* RNAi, either alone or combined with *AGO1* RNAi, significantly decreased *Myc* mRNA compared with control (*P*<0.0001 for both comparisons. (B) *Psi* qPCR for *AGO1* RNAi and/or *Psi* RNAi knockdown wing discs (20 discs pooled for each data point, mean±s.d. shown). Expression was performed for 2 days using *tub*-GAL4 in the background of *tub*-GAL80ts. AGO1 knockdown increased *Psi* mRNA compared with control (*P*=0.0002). *Psi* RNAi, either alone or combined with *AGO1* RNAi, significantly decreased *Psi* mRNA compared with control (*P*<0.0001 for both comparisons). (C) Anti-Myc antibody (purple) on *ser-GAL4*-driven *AGO1* RNAi compared with control. White asterisk indicates a region with elevated Myc protein in non-AGO1 knockdown cells. (D,E) qPCR for Myc-target genes *Polr1c* (D) and *CAD* (E) in wing discs following *AGO1* RNAi and/or *Psi* RNAi knockdown (20 discs pooled for each data point, mean±s.d. shown). Expression was performed for 2 days using *tub*-GAL4 in the background of *tub*-GAL80ts. AGO1 knockdown significantly increased *Polr1c* and *CAD* mRNA, compared with control (*P*=0.0005 and *P*=0.0043, respectively). Psi co-knockdown significantly reduced *Polr1c* and *CAD* mRNA compared with *AGO1* RNAi alone (*P*<0.0001 and *P*=0.0005 respectively). Myc co-knockdown significantly reduced *Polr1c* and *CAD* mRNA compared with *AGO1* RNAi alone (*P*=0.0003 and *P=*0.0205, respectively). Unpaired two-tailed *t*-test was used for all comparisons. NS, not significant; *P<0.05, **P<0.01, ***P<0.001 , ****P<0.0001.

The overgrowth observed in AGO1 knockdown wing imaginal disc cells (Figs 3,4) was associated with a significant increase in *Myc* mRNA, which was reduced by Psi co-knockdown (Fig. 5A). AGO1 knockdown also increased *Psi* mRNA (Fig. 5B) and protein abundance (Fig. S3), suggesting that AGO1 depletion might increase Myc (at least in part) by increasing abundance of Psi. Together, these data suggest that AGO1 represses *Myc* expression in larval wing discs in a manner partially dependent on Psi. In accordance with AGO1 normally being required for *Myc* repression, AGO1 depletion also increased Myc protein levels (Fig. 5C). To determine whether increased *Myc* mRNA and protein resulted in heightened Myc function (i.e. transcriptional activity) we monitored abundance of two established Myc target genes in mammalian and *Drosophila* systems, *Polr1c* (polymerase I polypeptide C) and *Cad* (carbamoyl-phosphate synthetase 2) (Mitchell et al., 2015; Poortinga et al., 2014, 2011). *Polr1c* and *Cad* mRNAs were significantly increased following AGO1 depletion, and co-knockdown of Psi or Myc reduced abundance of these Myc target mRNAs (Fig. 5D,E). Together, these data suggest that AGO1 is essential for restraining *Myc* levels and preventing cell overgrowth.

### Neither miR-996 nor miR-308 drive *Myc* mRNA turnover

Because AGO1 induces miRNA-dependent mRNA degradation as part of the RISC complex (Hutvagner and Simard, 2008), we tested whether AGO1 depletion altered *Myc* mRNA levels post transcriptionally. We screened miRBase (Griffiths-Jones, 2004), which contains published mature miRNA sequences from 223 species (Kozomara and Griffiths-Jones, 2014), for miRNAs predicted to target the *Myc* 3′UTR by sequence similarity (http://www.mirbase.org). miR-308 and miR-996 were the only miRNAs predicted to target *Myc* (Fig. S4A) that were also expressed in third instar larval tissues based on the modENCODE database (Contrino et al., 2012). In *Drosophila* embryos, miR-308 drives *Myc* mRNA and protein depletion (Daneshvar et al., 2013); however, overexpression of miR-308 did not reduce *Myc* mRNA in the larval wing imaginal disc (Fig. S4B**)**, suggesting that the capacity of miR-308 to target *Myc* depends on the developmental context. In contrast, miR-996 overexpression significantly increased *Myc* mRNA abundance (Fig. S4B), indicating that *Myc* mRNA is not a target for miR-996-driven degradation in the wing. Moreover, the capacity of *AGO1* knockdown to increase *Myc* was not altered by overexpression of either miR-308 or miR-996 (Fig. S4B), suggesting that AGO1 repression of *Myc* is not dependent on the function of either of the miRNAs predicted to target *Myc.*

### AGO1 protein localises to the cytoplasm and the nucleus

**Fig. 6.**
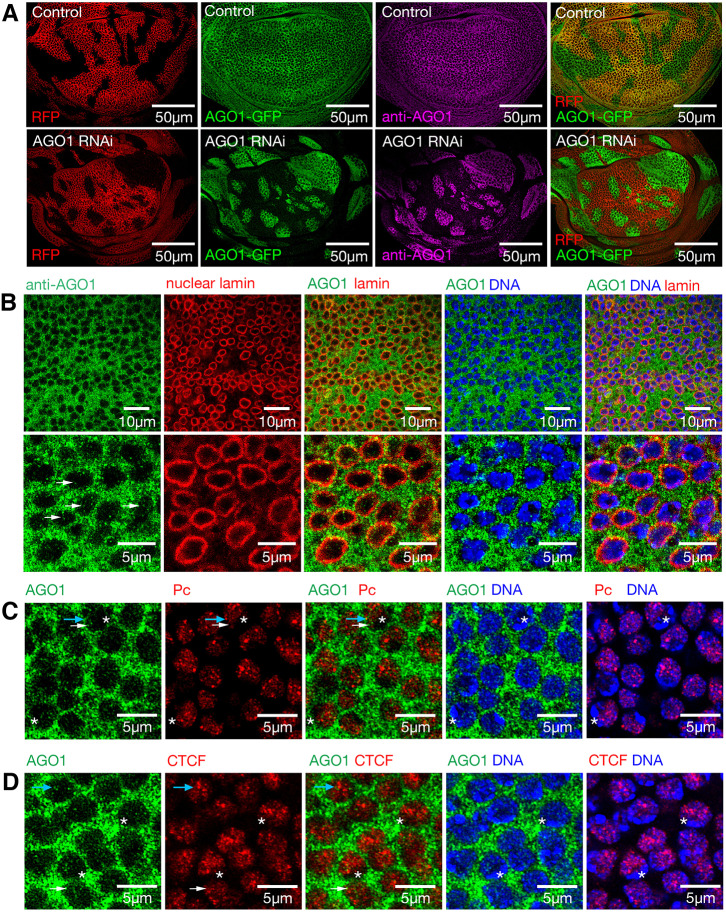
**AGO1 protein localises to both cytoplasmic and nuclear compartments.** (A) RFP-marked control and *AGO1* RNAi flip out clones in wing imaginal discs 2 days after heat shock in the AGO1-GFP protein trap background, stained with AGO1 antibody (purple). (B) Wild-type third instar wing imaginal discs stained with AGO1 antibody (green) and nuclear lamin (red). (C) Single 1 μm *z*-section from Zeiss Airyscan images for AGO1 antibody (green) and Pc-GFP (false coloured red). Blue arrow marks AGO1 puncta with PcG overlap, white arrow marks AGO1 without clear PcG overlap. Asterisks mark heterochromatin with intense DAPI staining. (D) AGO1 (green) and CTCF-GFP (false coloured red) for single 1 μm *z*-sections. Blue arrow marks three AGO1 puncta, the closest two without CTCF overlap and furthest one with overlap. White arrow marks AGO1 with weak CTCF overlap. Asterisks mark heterochromatin with intense DAPI staining.

AGO proteins, together with some components of the RISC, have been reported to enter the nucleus and regulate transcription (Catalanotto et al., 2016; Gosline et al., 2016; Kalantari et al., 2016; Shimada et al., 2016; Thomson et al., 2015; Woolnough et al., 2015). In early stage *Drosophila* blastoderm embryos, AGO1 protein localises to both the nucleus and cytoplasm (Pushpavalli et al., 2012). Biochemical fractionation and confocal immunofluorescence have also detected AGO proteins in the nuclear compartment of mammalian cells (Ahlenstiel et al., 2012). We therefore investigated the localisation of AGO1 in *Drosophila* wing imaginal disc cells using an anti-AGO1 antibody and an AGO1-GFP protein trap, which generates a GFP fusion with endogenous AGO1 (Buszczak et al., 2007). As expected, given miRNA silencing functions, AGO1 and the AGO1-GFP fusion localised predominantly to the cytoplasm in both the wing discs (Fig. 6A,B) and salivary glands (Fig. S5). In addition, co-staining with lamin to mark the nuclear envelope revealed punctate AGO1 staining within the nucleus.

As previous analysis in *Drosophila* antennal discs reported substantial overlap between AGO1 protein and Polycomb (Pc) body foci (42% colocalisation) (Grimaud et al., 2006), we examined whether AGO1 localises to regions of Polycomb group (PcG) mediated silencing in wing imaginal discs by staining with anti-AGO1 in the Pc-GFP background to mark PcG foci (Dietzel et al., 1999). In contrast to earlier studies using lower resolution microscopy, our high-resolution analysis separated PcG bodies from AGO1 puncta, revealing limited direct overlap (Fig. 6C). Indeed, quantification revealed overlap of just 8% and close proximity of 8.4% between AGO1 and PcG complexes, with the majority (83.6%) of staining occurring independently (Fig. S6A). To confirm that PcG bodies overlap euchromatin, as previously reported (Pirrotta and Li, 2012), DAPI was used to distinguish heterochromatin by intense staining, which revealed both PcG bodies and AGO1 puncta in regions of weaker DAPI staining; that is, AGO1 and PcG localise with euchromatin (Fig. 6C).

The observation that AGO1 puncta and PcG bodies localise to euchromatin, but generally do not directly overlap (83.6%), lends support to the idea that multiprotein and RNA complexes containing AGO1 might serve as a scaffold for assembly of the PcG supercomplexes that underlie both PcG and insulator bodies (Pirrotta and Li, 2012; Shevtsov and Dundr, 2011). Although AGO2 has been reported to enable insulator function independent of RNAi activity through physical association with CTCF binding sites in *Drosophila* (Moshkovich et al., 2011), such roles have not been reported for AGO1. We therefore tested proximity between AGO1 and chromatin insulator bodies, and the localisation of AGO1 and CTCF chromatin insulator complexes in the nucleus using anti-AGO1 and CTCF-GFP (Fig. 6D). As expected, based on DAPI staining, AGO1 complexes were detected in regions of euchromatin; however, only 15% of the AGO1 puncta were found overlapping or in close proximity with CTCF-marked insulator domains (Fig. 6D; quantified in Fig. S6B). Together these data suggest that AGO1 complexes interact with a small subpopulation of PcG transcriptional silencing loci and CTCF insulator domains in the nucleus.

### AGO1 knockdown increases *Myc* transcription

**Fig. 7.**
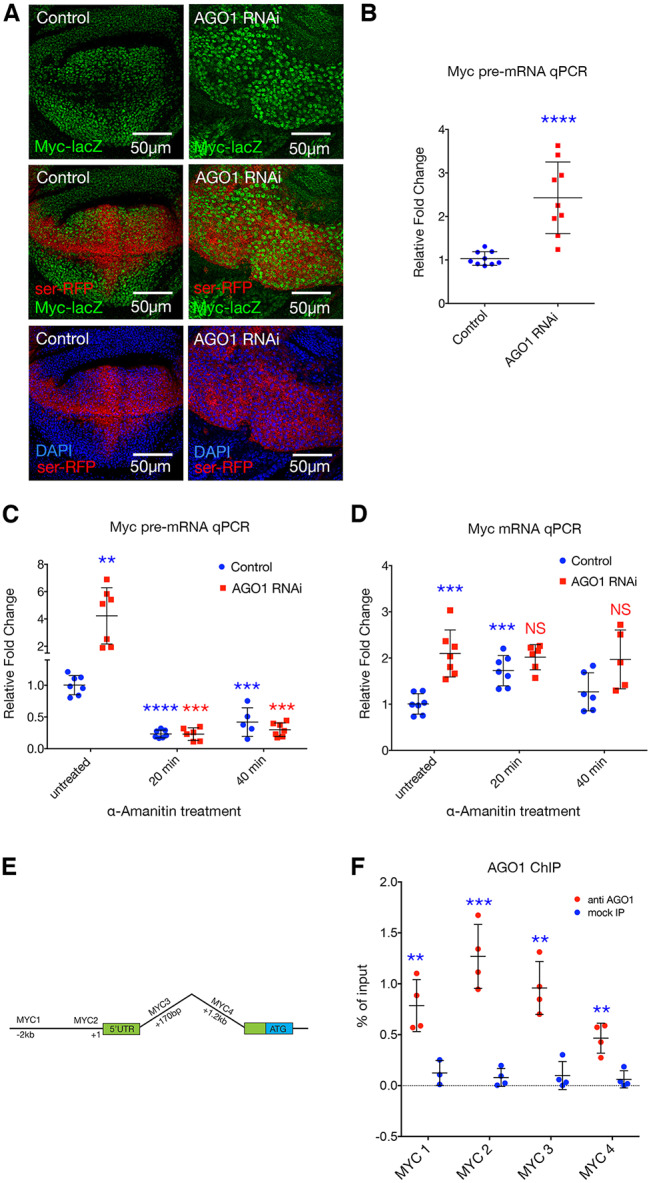
**AGO1 represses *Myc* at the level of transcription.** (A) *Myc-lacZ* enhancer trap activity, marked with anti-β-Gal antibody (green) for *ser*-GAL4-driven *AGO1* RNAi in the *UAS-*p35 background compared with *UAS-p35* alone control, marked with RFP and stained for DNA (blue). (B) qPCR for *Myc* pre-mRNA following *AGO1* RNAi knockdown in larval wing discs (20 discs pooled for each data point, mean±s.d. shown). Expression was performed for 2 days using *tub*-GAL4 in the background of *tub*-GAL80ts. *AGO1* RNAi was compared with control. (C) qPCR for *Myc* pre-mRNA following AGO1 knockdown compared with control, performed for 2 days using *tub*-GAL4 in the background of *tub*-GAL80ts, in larval head tissues treated with α-amanitin for 20 or 40 min or untreated (five heads pooled for each data point, mean±s.d. shown). *P*=0.0014 for untreated *AGO1* RNAi compared with untreated control. *P*<0.0001 for 20 min α-amanitin-treated control compared with untreated control. *P*=0.0003 for 40 min α-amanitin-treated control compared with untreated control. *P*=0.0006 for *AGO1* RNAi 20 min α-amanitin-treated compared with *AGO1* RNAi untreated. *P*=0.0003 for *AGO1* RNAi 40 min α-amanitin-treated compared with *AGO1* RNAi untreated. (D) qPCR for mature *Myc* mRNA; genotypes and treatment with α-amanitin as described in C. *P*=0.0002 for untreated *AGO1* RNAi compared with untreated control. *P*=0.0004 for 20 min α-amanitin treated control compared with untreated control. (E) Schematic of *Myc* showing the position of amplicons used for qPCR. (F) AGO1 ChIP on wild-type larval tissues compared with no-antibody (mock IP) controls (*n*=4 independent experiments, mean±s.d. shown). *P*=0.0095 at MYC1, *P*=0.0003 at MYC2, *P*=0.0011 at MYC3 and *P*=0.0031 at MYC4 for AGO1 antibody compared with control. Unpaired two-tailed *t*-test was used for all comparisons. NS, not significant; **P<0.01, ***P<0.001, ****P<0.0001.

Recent studies demonstrated transcriptional regulation of the *MYC* oncogene involving looping of super-enhancers and that the *MYC* promoter requires a conserved CTCF site (Schuijers et al., 2018). This, together with our observations that AGO1 interacts with the RNA Pol II machinery, localises to euchromatic regions of DNA and overlaps PcG and CTCF complexes, led us to investigate whether AGO1 regulates *Myc* mRNA abundance at the level of transcription. Indeed, AGO1 is required to constrain the *Myc* promoter, as *Myc-lacZ* enhancer trap (Mitchell et al., 2010; Peter et al., 2002) activity was significantly increased in the *AGO1* knockdown wing disc compartment (Fig. 7A). To further investigate whether increased *Myc* mRNA associated with AGO1 loss of function was caused by altered transcription, we designed primers to the first intron of *Myc* to measure pre-mRNA levels. Quantitative PCR revealed an increase in the immature *Myc* message following AGO1 knockdown in wing discs (Fig. 7B). Together, these data suggest that the increased *Myc* expression associated with AGO1 depletion is a result of activation of the *Myc* promoter and increased transcription.

### Increased *Myc* due to AGO1 depletion requires RNA Pol II transcription

To determine whether AGO1 regulates *Myc* expression at the transcriptional level, we used α-amanitin to block RNA Pol II activity. Consistent with observations using wing imaginal discs (Fig. 5), *Myc* pre- and processed mRNA levels were significantly increased in untreated larval head tissues following AGO1 depletion (Fig. 7C,D). Interestingly, although *Myc* pre-mRNA was significantly decreased 20 and 40 min after α-amanitin treatment (Fig. 7C), mature mRNA was significantly increased in control tissues at the 20 min time point (Fig. 7D), suggesting that *Myc* mRNA stability might increase in response to transcriptional inhibition. In the AGO1 knockdown background, *Myc* pre-mRNA levels were dramatically decreased following α-amanitin treatment (Fig. 7C). Thus, the AGO1 knockdown-induced increase in *Myc* pre-mRNA was dependent on RNA Pol II transcriptional activity. Abundance of the mature *Myc* transcript was not significantly altered in the AGO1 knockdown background following α-amanitin treatment, again suggesting that feedback mechanisms might result in increased mRNA stability in response to RNA Pol II inhibition. Together with the observation that AGO1 knockdown increased *Myc* promoter activity in the wing discs, these data suggest that AGO1 is normally required for repression of *Myc* transcription.

### AGO1 is enriched on *Myc*

The increased *Myc* promoter activity following AGO1 depletion, and increased *Myc* mRNA abundance requiring RNA Pol II activity, led us to test whether AGO1 directly regulates *Myc* transcription. We therefore performed ChIP using the anti-AGO1 antibody followed by qPCR with amplicons flanking the *Myc* transcription start site (Fig. 7E). In wild-type larval tissues, significant AGO1 enrichment was observed in *Myc* regulatory regions compared with the mock-IP control, with highest enrichment observed at the transcription start site (Fig. 7F), suggesting that AGO1 normally inhibits *Myc* transcription through direct interaction with the *Myc* promoter.

## DISCUSSION

Here, we demonstrate a novel role for AGO1 as a growth inhibitor in *Drosophila.* AGO1 depletion was sufficient to increase Myc (mRNA and protein) to drive ribosome biogenesis, nucleolar expansion and cell growth in a Myc and Psi-dependent manner. The increased *Myc* promoter activity in AGO1 knockdown wing discs, together with the α-amanitin-dependent increase in *Myc* pre-mRNA abundance, suggests that AGO1 represses *Myc* at the level of transcription. In accordance with the observed growth inhibitory capacity of AGO1, the increased *Myc* mRNA and protein abundance in AGO1 knockdown wings were associated with increased Myc function (i.e. activation of established Myc targets). Interestingly, although Psi co-knockdown only modestly decreased *Myc* mRNA levels in AGO1-depleted wings, Psi co-depletion strongly reduced expression of Myc targets. This observation suggests that Psi is not only required for *Myc* transcription (Guo et al., 2016) but may also be required for activation of Myc growth targets in the context of AGO1 depletion. Thus, future studies are required to determine whether Psi and Myc bind common targets and whether Psi is required for transcriptional activation of Myc target genes.

Recent genome-wide functional RNAi screens in *Drosophila* S2 cells, identifying AGO1 as a modifier of Polycomb foci, suggested extra-miRNA functions for AGO1 (Gonzalez et al., 2014). PcG mediates epigenetic repression of key developmental genes to control cell fate, and PcG repression is stabilised via aggregation of PcG foci in the nucleus. AGO1 depletion disrupted nuclear organisation and reduced the intensity of Pc foci, suggesting that AGO1 negatively regulates PcG-mediated silencing (Grimaud et al., 2006). The *Drosophila* PcG complex has been characterised for roles in silencing homeotic genes by binding PcG response elements (PREs), including the Fab-7 PRE-containing regulatory element from the Hox gene, Abdominal-B. Components of the RNAi machinery, including AGO1 and Dicer-2, have been implicated in driving PcG-dependent silencing between remote copies of the Fab-7 element, engineered throughout the genome to monitor long-distance gene contacts. Interactions between Hox genes silenced by PcG proteins were decreased in *AGO1* mutants, suggesting that AGO1 regulates nuclear organisation, at least in part, by stabilising PcG protein recruitment to chromatin (Grimaud et al., 2006).

*Myc* transcriptional autorepression, modelled in the *Drosophila* embryo via overexpression of *Myc* from an exogenous promoter, leads to repression of the endogenous *Myc* locus in a Pc-dependent manner (Goodliffe et al., 2005). This, together with the partial overlap between AGO1 and PcG in wing imaginal disc cells (Fig. 6C), suggests that Pc mediates transcriptional autorepression of *Myc* via AGO1. In contrast to our studies, where AGO1 depletion phenotypes are associated with a moderate (>three- to fivefold) increase in Myc, autoregulation in the embryo was investigated in response to non-physiological increases in Myc (over 100 times endogenous levels) (Goodliffe et al., 2005). Thus, our data suggest that AGO1 binds the *Myc* promoter under normal conditions and is required for repression of endogenous *Myc* transcription (Fig. 5A), but whether AGO1 is required for Pc-dependent *Myc* autorepression requires further investigation. In a similar vein, super-enhancers control human *MYC* transcription via CTCF in the context of high-MYC cancers (Schuijers et al., 2018). Thus, failed Pc-dependent autorepression and/or defective repression of super-enhancers via CTCF could further elevate MYC to promote cancer progression. Given the observed overlap between AGO1 and Pc/CTCF in the *Drosophila* wing, future studies determining whether AGO1 interacts with Pc and/or CTCF to control autoregulatory feedback on *Myc* transcription in the context of tumorigenesis will be of great interest.

The question remains regarding how AGO1 targets *Myc* transcription. The physical and genetic interaction between Psi and AGO1, and the observation that AGO1 loss-of-function mutants restore cell and tissue growth in the Psi knockdown wing, suggests that AGO1 inhibits growth that is dependent on this Myc transcriptional regulator. AGO2 has been implicated in insulator-dependent looping interactions defining 3D transcriptional domains (TADs) through association with CTCF binding sites in *Drosophila* (Moshkovich et al., 2011). Although similar roles for AGO1 have not been reported, the cancer-related super-enhancers for the *MYC* oncogene lie within the 2.8 Mb TAD and control *MYC* transcription via a common and conserved CTCF binding site located 2 kb upstream of the *MYC* promoter; that is, in proximity with the FUSE (1.7 kb upstream) bound by FUBP1. Moreover, gene disruption of the enhancer-docking site reduces CTCF binding and super-enhancer interaction, which results in reduced *MYC* expression and proliferative cell growth (Schuijers et al., 2018). AGO1 ChIP revealed significant enrichment on the *Myc* promoter, suggesting that AGO1 probably interacts with Psi and the RNA Pol II machinery to directly regulate *Myc* transcription. Given the high level of conservation between AGO and CTCF proteins throughout evolution, it is of great interest to determine whether human AGO1 also interacts with FUBP1 to regulate transcription of the *MYC* oncogene.

Here, we have shown that AGO1 behaves as a growth inhibitor during *Drosophila* development, through the ability to suppress *Myc* transcription, ribosome biogenesis and cell growth in the wing disc epithelium. Consistent with AGO1 having tumour suppressor activity, across a wide range of human cancers, large scale genomics data in cBioPortal (Gao et al., 2013) identified AGO1 as frequently mutated or deleted in a diverse variety of tumours (e.g. reproductive, breast, intestinal, bladder, and skin cancers). Region 1p34–35 of chromosome 1, which includes AGO1, is frequently deleted in Wilms' tumours and neuro-ectodermal tumours (Parisi et al., 2011). In neuroblastoma cell lines, AGO1 behaves as a tumour suppressor, with overexpression heightening checkpoint sensitivity and reducing cell cycle progression. GEO Profile microarray data inversely correlates AGO1 expression with proliferative index (Parisi et al., 2011); that is, AGO1 levels are significantly lower in tumorigenic cells than in differentiated cells (Barrett et al., 2011). In the context of cancer, it is important to determine whether AGO1 loss of function alters *MYC-*dependent cancer progression and vice versa. As increased abundance of the MYC oncoprotein is associated with the pathogenesis of most human tumours (Dang, 2012; Levens, 2010), deciphering such novel mechanisms of *MYC* repression is fundamental to understanding MYC-dependent cancer initiation and progression.

## MATERIALS AND METHODS

### *Drosophila* strains

The *UAS-AGO1* RNAi 1 (BL53293), *UAS-AGO1* RNAi 2 (BL33727), *UAS-Myc* (BL9675), AGO-GFP (BL50805), *UAS-miR-308* (BL41809), *UAS*-*miR-996* (BL60653), *Myc-lacZ* (BL12247), CTCF-GFP (BL64810), Pc-GFP (BL9593), *ser-*GAL4 (BL6791), *tub*-GAL4 (BL5138), *tub*-GAL80ts (BL7019), *hsflp* (BL1929) and *Act*<CD2<GAL4 (BL30558) were obtained from the Bloomington *Drosophila* Stock Center. The *UAS-Myc* RNAi (V2947), *UAS-Psi* RNAi (V105135), *AGO1^k00208^* (V10470) and *AGO1^04845^* (V11388) lines were obtained from the Vienna *Drosophila* RNAi Center.

### Co-immunoprecipitation and western blotting

Co-IP was performed using 25 wild-type third instar larval heads dissociated in cold lysis buffer (50 mM Tris pH 7.5, 1.5 mM MgCl_2_, 125 mM NaCl, 0.2% NP40, 5% glycerol, 1× protease inhibitor cocktail). Following homogenisation, protein was collected by centrifugation at 13,000 ***g*** for 10 min at 4°C. The extract was pre-cleared by incubation with nProtein A Sepharose beads (GE Healthcare Life Science) for 1 h at 4°C with rotation and the supernatant collected by centrifugation at 13,000 ***g***. Equal amounts of pre-cleared protein lysate were incubated with either anti-AGO1 antibody (Abcam, ab5070, 1:70), anti-Psi antibody (custom generated rabbit polyclonal antibody, Biomatik, 1:100) or without antibody (mock IP control) overnight at 4°C. Beads were washed five times with lysis buffer and the eluent resolved using 10% SDS PAGE/western blot with appropriate primary antibody before detection with Li-Cor Odyssey IR.

### Immunofluorescence, microscopy and image analysis

Because of the high level of cell death and lethality associated with constitutive *ser*-GAL4-driven AGO1 depletion, analysis was conducted with co-expressed baculoviral caspase inhibitor p35 to prevent apoptosis (Hay et al., 1994); crosses were maintained at 18°C, unless otherwise stated. Wandering third instar larvae were dissected and fixed for 20 min in 4% paraformaldehyde (PFA), washed in PBS containing 0.1% Tween (PBT), blocked in 5 mg/ml bovine serum albumin (BSA) prior to incubation overnight at 4°C with primary antibody. Primary antibodies used for immunofluorescence were Myc N (rabbit, 1:500; Santa Cruz d46-507), fibrillarin (rabbit, 1:500; Abcam ab5821), AGO1 (rabbit, 1:500; Abcam ab5070), Psi (rabbit, 1:500; custom-made via Biomatik), Lamin (mouse, 1:20; DSHB ADL 101), β-galactosidase (chicken, 1:1000; Abcam ab9361). After incubating with appropriate fluorophore-tagged secondary antibodies (Jackson ImmunoResearch Laboratories; anti-rabbit 488, 1:1000, 711-545-152; anti-chicken 488, 1:1000, 703-485-155; anti-mouse 649, 1:1000, 715-495-151; and anti-rabbit 647, 1:1000, 711-605-152), samples were counterstained with DAPI solution and wing imaginal discs imaged using a Zeiss LSM800 confocal microscope (Zen Blue software). Overlapping 1 μm *z*-sections were collected at 40× magnification. Fluorophores were imaged using band-pass filters to remove cross-detection between channels. Images were processed and prepared using ImageJ and Adobe Photoshop CS5. Fibrillarin- and CD8-RFP-marked cell sizes were quantified in FIJI on confocal *z*-sections of wing columnar epithelial cells, merged to display maximum projections (two or three sections). Thresholding was performed and images were used to measure average fibrillarin area or cell size in the dorsal compartment marked by *serrate-*GAL4>*UAS*-RFP expression. About 50-100 nucleoli were selected using the freeform selection tool and analysed with the ‘Analyse Particles’ tool, with minimum particle size of 0.5 μm^2^ applied in order to exclude noise and out-of-focus nucleoli. For cell size, 50-100 cells per wing disc were analysed, with a minimum particle size of 3 μm^2^ applied. The output used image metadata to calculate average area for each wing disc analysed. Analysis of percentage overlap between AGO1 and PcG/CTCF was performed in FIJI by thresholding to isolate individual puncta and overlaying channels to detect co-occurrence or adjacency, which was counted and expressed as a proportion of total AGO1 puncta per individual nuclei.

### Adult wing size analysis

Adult wing size was determined for male wings imaged with an Olympus SZ51 binocular microscope, at 4.5× magnification using the Olympus DP20 camera. Wing size was measured by pixel count for the area posterior to wing vein L5, using Photoshop CS5. For wing hair counts, adult male wings were imaged with an Olympus BX 61 microscope at 20× magnification using the Olympus DP70 camera. Wing cell size was determined using wing hair counts in a defined area (200×100 pixels) at the central region posterior to wing vein L5. Then, the hair number was converted to relative single cell size by dividing the area of the fixed region by hair numbers.

### Quantitative PCR

qPCR was performed 2 days after induction of transgene expression using *tub*-GAL4 in *tub*-GAL80ts background. RNA was isolated from equivalent numbers of wing imaginal discs (10 pairs for each genotype) using the Promega ReliaPrep RNA Cell miniprep system and eluted in 20 μl nuclease-free water. RNA purity and integrity were assessed using an automated electrophoresis system (2200 TapeStation, Agilent Technologies). Each cDNA synthesis used 6 μl of RNA (GoScript Reverse Transcription System kit, Promega). qPCR was performed using Fast SYBR Green Master Mix (Applied Biosystems) using the StepOnePlus Real-Time PCR System and Sequence Detection Systems in 96-well plates (Applied Biosystems; 95°C for 2 min, 40 cycles 95°C 1 s and 60°C 20 s). Amplicon specificity was verified by melt curve analysis. Average Ct values for two technical replicates were calculated for each sample. Multiple candidate reference genes were analysed for stability across *AGO1* RNAi and control samples using RefFinder. Target gene expression was normalized to the mean of *cyp1* and *tubulin*, selected for having high expression and smallest sample-to-sample variability. Fold change was determined using the 2-ΔΔCT method.

### Primers used for qPCR

Myc, 5′-GTGGACGATGGTCCCAATTT-3′ and 5′-GGGATTTGTGGGTAGCTTCTT-3′; Myc pre-mRNA, 5′-TTCAAAATAGAATTTCTGGGAAAGGT-3′ and 5′-GCGGCCATGATCACTGATT-3′; Psi, 5′-CGATGGCATCCCATTTGTTTGT-3′ and 5′-GGTGGTCAAGACTACTCGGC-3′; AGO1, 5′-ACTCTACGGTCTGTCCGTTC-3′ and 5′-CCCGCTCAGATGCAATCATTC-3′; 5′-ETS, 5′-GGCAGTGGTTGCCGACCTCG-3′ and 5′-GCGGAGCCAAGTCCCGTGTT-3′; Tubulin, 5′-TGGGCCCGTCTGGACCACAA-3′ and 5′-TCGCCGTCACCGGAGTCCAT-3′; CYP1, 5′-TCGGCAGCGGCATTTCAGAT-3′ and 5′-TGCACGCTGACGAAGCTAGG-3′; Polr1c, 5′-TGTATCCCGCCATTGCAA-3′ and 5′-GGGCACATCGCTGAGCAT-3′; Cad, 5′-CATTGGCAGTTTCAAGCACAA-3′ and 5′-TCTTGGCCAGATCCCGTATG-3′.

### α-Amanitin treatment

α-Amanitin inhibits RNA Pol II-dependent transcription, therefore interfering with mRNA production (Lindell et al., 1970). The α-amanitin (Sigma #A2263) was diluted in 1 ml of Nanopure water to make a 1 mg/ml stock solution, which was stored at −20°C in the dark. Samples were collected 2 days after induction of transgene expression using *tub*-GAL4 in *tub*-GAL80ts background. Third instar larval heads (anterior imaginal tissues) were dissected and incubated with freshly made 20 μg/ml α-amanitin in Schneider's medium at 25°C for 0, 20 or 40 min. After α-amanitin treatment, samples were washed for 5 min using fresh Schneider's medium and snap frozen in 250 μl LBA+TG lysis buffer from the Promega ReliaPrep RNA Cell Miniprep kit. Following RNA extraction and cDNA synthesis, qPCR was performed and analysed with *Myc* cDNA primers and *Myc* pre-mRNA primers.

### Chromatin immunoprecipitation

ChIP assays were carried out as described previously (Lee et al., 2015). Briefly, for each ChIP sample, 30 larval heads were collected from wild-type mid-third instar larvae and fixed in 4% PFA for 20 min. Larval heads were dissociated and chromatin sheared in 0.4% sodium dodecyl sulphate (SDS) using a Covaris S2 (10 min duration, 10% DUTY, 200 cycles per burst, intensity 4, achieving average DNA fragment sizes of 200-600 bp). ChIP was performed in IP buffer containing 0.1% SDS and 3 µg of anti-RNA Pol II phospho-S5 antibody (Abcam, ab5131) or anti-RNA Pol II phospho-S2 (Abcam, ab5095) for each IP. As a control, mock IP was performed without the use of an antibody. Analysis was performed in triplicate using Fast SYBR Green Master Mix (Applied Biosystems) on a StepOnePlus Real-Time PCR System and Sequence Detection Systems in 384-well plates (Applied Biosystems). To calculate the percentage of total DNA bound, non-immunoprecipitated input samples from each condition were used as the qPCR reference for all qPCR reactions.

### Primers for ChIP qPCR

The following primers were used for ChIP qPCR: MYC 1, 5′-GGCGATCGTTTCTGGCCTACGG-3′ and 5′-GCAGGCGCATTTGACTCGGC-3′; MYC 2, 5′-ACTACTACTAACAACTGTCACAAGCCAAGT-3′ and 5′-TTTATGTATTTGCGCGGTTTTAAG-3′; MYC 3, 5′-TTCAAAATAGAATTTCTGGGAAAGGT-3′ and 5′-GCGGCCATGATCACTGATT-3′; MYC 4, 5′-GGTTTTCCTTTTATGCCCTTG-3′ and 5′-CTATTAACCATTTGAACCCGAAATC-3′.

### Statistical analysis

All statistical tests were performed with Graphpad Prism 6 using an unpaired two-tailed *t*-test with 95% confidence interval. In all figures, error bars represent s.d. and, according to the Graphpad classification of significance points, *P=0.01-0.05, **P=0.001-0.01, ***P=0.0001-0.001 and ****P<0.0001.

## Supplementary Material

Supplementary information

Reviewer comments

## References

[DEV190231C1] Ahlenstiel C. L., Lim H. G. W., Cooper D. A., Ishida T., Kelleher A. D. and Suzuki K. (2012). Direct evidence of nuclear Argonaute distribution during transcriptional silencing links the actin cytoskeleton to nuclear RNAi machinery in human cells. *Nucleic Acids Res.* 40, 1579-1595. 10.1093/nar/gkr891 22064859 PMC3287199

[DEV190231C2] Arabi A., Wu S., Ridderstråle K., Bierhoff H., Shiue C., Fatyol K., Fahlén S., Hydbring P., Söderberg O., Grummt I. et al. (2005). c-Myc associates with ribosomal DNA and activates RNA polymerase I transcription. *Nat. Cell Biol.* 7, 303-310. 10.1038/ncb1225 15723053

[DEV190231C3] Atwood B. L., Woolnough J. L., Lefevre G. M., Saint Just Ribeiro M., Felsenfeld G. and Giles K. E. (2016). Human Argonaute 2 is tethered to ribosomal RNA through MicroRNA interactions. *J. Biol. Chem.* 291, 17919-17928. 10.1074/jbc.M116.725051 27288410 PMC5016180

[DEV190231C4] Barrett T., Troup D. B., Wilhite S. E., Ledoux P., Evangelista C., Kim I. F., Tomashevsky M., Marshall K. A., Phillippy K. H., Sherman P. M. et al. (2011). NCBI GEO: archive for functional genomics data sets--10 years on. *Nucleic Acids Res.* 39, D1005-D1010. 10.1093/nar/gkq1184 21097893 PMC3013736

[DEV190231C5] Buszczak M., Paterno S., Lighthouse D., Bachman J., Planck J., Owen S., Skora A. D., Nystul T. G., Ohlstein B., Allen A. et al. (2007). The carnegie protein trap library: a versatile tool for Drosophila developmental studies. *Genetics* 175, 1505-1531. 10.1534/genetics.106.065961 17194782 PMC1840051

[DEV190231C6] Catalanotto C., Cogoni C. and Zardo G. (2016). MicroRNA in control of gene expression: an overview of nuclear functions. *Int. J. Mol. Sci.* 17, 1712 10.3390/ijms17101712 PMC5085744 27754357

[DEV190231C7] Challagundla K. B., Sun X.-X., Zhang X., DeVine T., Zhang Q., Sears R. C. and Dai M.-S. (2011). Ribosomal protein L11 recruits miR-24/miRISC to repress c-Myc expression in response to ribosomal stress. *Mol. Cell. Biol.* 31, 4007-4021. 10.1128/MCB.05810-11 21807902 PMC3187350

[DEV190231C8] Chung H. J. and Levens D. (2005). c-myc expression: keep the noise down! *Mol. Cells* 20, 157-166. 16267388

[DEV190231C9] Chung H.-J., Liu J., Dundr M., Nie Z., Sanford S. and Levens D. (2006). FBPs are calibrated molecular tools to adjust gene expression. *Mol. Cell. Biol.* 26, 6584-6597. 10.1128/MCB.00754-06 16914741 PMC1592819

[DEV190231C10] Contrino S., Smith R. N., Butano D., Carr A., Hu F., Lyne R., Rutherford K., Kalderimis A., Sullivan J., Carbon S. et al. (2012). modMine: flexible access to modENCODE data. *Nucleic Acids Res.* 40, D1082-D1088. 10.1093/nar/gkr921 22080565 PMC3245176

[DEV190231C11] Dalla-Favera R., Bregni M., Erikson J., Patterson D., Gallo R. C. and Croce C. M. (1982). Human c-myc onc gene is located on the region of chromosome 8 that is translocated in Burkitt lymphoma cells. *Proc. Natl. Acad. Sci. USA* 79, 7824-7827. 10.1073/pnas.79.24.7824 6961453 PMC347441

[DEV190231C12] Daneshvar K., Nath S., Khan A., Shover W., Richardson C. and Goodliffe J. M. (2013). MicroRNA miR-308 regulates dMyc through a negative feedback loop in Drosophila. *Biol. Open* 2, 1-9. 10.1242/bio.20122725 23336071 PMC3545263

[DEV190231C13] Dang C. V. (2010). Enigmatic MYC conducts an unfolding systems biology symphony. *Genes Cancer* 1, 526-531. 10.1177/1947601910378742 21218193 PMC3017351

[DEV190231C14] Dang C. V. (2012). MYC on the path to cancer. *Cell* 149, 22-35. 10.1016/j.cell.2012.03.003 22464321 PMC3345192

[DEV190231C15] Dietzel S., Niemann H., Brückner B., Maurange C. and Paro R. (1999). The nuclear distribution of Polycomb during Drosophila melanogaster development shown with a GFP fusion protein. *Chromosoma* 108, 83-94. 10.1007/s004120050355 10382070

[DEV190231C16] Fernandez P. C., Frank S. R., Wang L., Schroeder M., Liu S., Greene J., Cocito A. and Amati B. (2003). Genomic targets of the human c-Myc protein. *Genes Dev.* 17, 1115-1129. 10.1101/gad.1067003 12695333 PMC196049

[DEV190231C17] Förstemann K., Horwich M. D., Wee L. M., Tomari Y. and Zamore P. D. (2007). Drosophila microRNAs are sorted into functionally distinct argonaute complexes after production by dicer-1. *Cell* 130, 287-297. 10.1016/j.cell.2007.05.056 17662943 PMC2686109

[DEV190231C18] Gao J., Aksoy B. A., Dogrusoz U., Dresdner G., Gross B., Sumer S. O., Sun Y., Jacobsen A., Sinha R., Larsson E. et al. (2013). Integrative analysis of complex cancer genomics and clinical profiles using the cBioPortal. *Sci. Signal.* 6, pl1 10.1126/scisignal.2004088 23550210 PMC4160307

[DEV190231C19] Gomez-Roman N., Felton-Edkins Z. A., Kenneth N. S., Goodfellow S. J., Athineos D., Zhang J., Ramsbottom B. A., Innes F., Kantidakis T., Kerr E. R. et al. (2006). Activation by c-Myc of transcription by RNA polymerases I, II and III. *Biochem. Soc. Symp.* 73, 141-154. 10.1042/bss0730141 16626295

[DEV190231C20] Gonzalez I., Mateos-Langerak J., Thomas A., Cheutin T. and Cavalli G. (2014). Identification of regulators of the three-dimensional polycomb organization by a microscopy-based genome-wide RNAi screen. *Mol. Cell* 54, 485-499. 10.1016/j.molcel.2014.03.004 24703951

[DEV190231C21] Goodliffe J. M., Wieschaus E. and Cole M. D. (2005). Polycomb mediates Myc autorepression and its transcriptional control of many loci in Drosophila. *Genes Dev.* 19, 2941-2946. 10.1101/gad.1352305 16357214 PMC1315398

[DEV190231C22] Gosline S. J. C., Gurtan A. M., JnBaptiste C. K., Bosson A., Milani P., Dalin S., Matthews B. J., Yap Y. S., Sharp P. A. and Fraenkel E. (2016). Elucidating MicroRNA regulatory networks using transcriptional, post-transcriptional, and Histone modification measurements. *Cell Rep.* 14, 310-319. 10.1016/j.celrep.2015.12.031 26748710 PMC4831719

[DEV190231C23] Grandori C., Gomez-Roman N., Felton-Edkins Z. A., Ngouenet C., Galloway D. A., Eisenman R. N. and White R. J. (2005). c-Myc binds to human ribosomal DNA and stimulates transcription of rRNA genes by RNA polymerase I. *Nat. Cell Biol.* 7, 311-318. 10.1038/ncb1224 15723054

[DEV190231C24] Grewal S. S., Li L., Orian A., Eisenman R. N. and Edgar B. A. (2005). Myc-dependent regulation of ribosomal RNA synthesis during Drosophila development. *Nat. Cell Biol.* 7, 295-302. 10.1038/ncb1223 15723055

[DEV190231C25] Griffiths-Jones S. (2004). The microRNA registry. *Nucleic Acids Res.* 32, D109-D111. 10.1093/nar/gkh023 14681370 PMC308757

[DEV190231C26] Grimaud C., Bantignies F., Pal-Bhadra M., Ghana P., Bhadra U. and Cavalli G. (2006). RNAi components are required for nuclear clustering of Polycomb group response elements. *Cell* 124, 957-971. 10.1016/j.cell.2006.01.036 16530043

[DEV190231C27] Guo L., Zaysteva O., Nie Z., Mitchell N. C., Amanda Lee J. E., Ware T., Parsons L., Luwor R., Poortinga G., Hannan R. D. et al. (2016). Defining the essential function of FBP/KSRP proteins: Drosophila Psi interacts with the mediator complex to modulate MYC transcription and tissue growth. *Nucleic Acids Res.* 44, 7646-7658. 10.1093/nar/gkw461 27207882 PMC5027480

[DEV190231C28] Guruharsha K. G., Rual J.-F., Zhai B., Mintseris J., Vaidya P., Vaidya N., Beekman C., Wong C., Rhee D. Y., Cenaj O. et al. (2011). A protein complex network of Drosophila melanogaster. *Cell* 147, 690-703. 10.1016/j.cell.2011.08.047 22036573 PMC3319048

[DEV190231C29] Hay B. A., Wolff T. and Rubin G. M. (1994). Expression of baculovirus P35 prevents cell death in Drosophila. *Development* 120, 2121-2129. 7925015 10.1242/dev.120.8.2121

[DEV190231C30] He L., Liu J., Collins I., Sanford S., O'Connell B., Benham C. J. and Levens D. (2000). Loss of FBP function arrests cellular proliferation and extinguishes c-myc expression. *EMBO J.* 19, 1034-1044. 10.1093/emboj/19.5.1034 10698944 PMC305642

[DEV190231C31] Hutvagner G. and Simard M. J. (2008). Argonaute proteins: key players in RNA silencing. *Nat. Rev. Mol. Cell Biol.* 9, 22-32. 10.1038/nrm2321 18073770

[DEV190231C32] Kalantari R., Chiang C.-M. and Corey D. R. (2016). Regulation of mammalian transcription and splicing by Nuclear RNAi. *Nucleic Acids Res.* 44, 524-537. 10.1093/nar/gkv1305 26612865 PMC4737150

[DEV190231C33] Kozomara A. and Griffiths-Jones S. (2014). miRBase: annotating high confidence microRNAs using deep sequencing data. *Nucleic Acids Res.* 42, D68-D73. 10.1093/nar/gkt1181 24275495 PMC3965103

[DEV190231C34] Labourier E., Adams M. D. and Rio D. C. (2001). Modulation of P-element pre-mRNA splicing by a direct interaction between PSI and U1 snRNP 70K protein. *Mol. Cell* 8, 363-373. 10.1016/S1097-2765(01)00311-2 11545738

[DEV190231C35] Lee J. E. A., Mitchell N. C., Zaytseva O., Chahal A., Mendis P., Cartier-Michaud A., Parsons L. M., Poortinga G., Levens D. L., Hannan R. D. et al. (2015). Defective Hfp-dependent transcriptional repression of dMYC is fundamental to tissue overgrowth in Drosophila XPB models. *Nat. Commun.* 6, 7404 10.1038/ncomms8404 26074141 PMC7720677

[DEV190231C36] Levens D. (2010). You Don't Muck with MYC. *Genes Cancer* 1, 547-554. 10.1177/1947601910377492 20882108 PMC2946075

[DEV190231C37] Liao D. J. and Dickson R. B. (2000). c-Myc in breast cancer. *Endocr. Relat. Cancer* 7, 143-164. 10.1677/erc.0.0070143 11021963

[DEV190231C38] Lindell T. J., Weinberg F., Morris P. W., Roeder R. G. and Rutter W. J. (1970). Specific inhibition of nuclear RNA polymerase II by alpha-amanitin. *Science* 170, 447-449. 10.1126/science.170.3956.447 4918258

[DEV190231C39] Liu J., Kouzine F., Nie Z., Chung H.-J., Elisha-Feil Z., Weber A., Zhao K. and Levens D. (2006). The FUSE/FBP/FIR/TFIIH system is a molecular machine programming a pulse of c-myc expression. *EMBO J.* 25, 2119-2130. 10.1038/sj.emboj.7601101 16628215 PMC1462968

[DEV190231C40] Marcu K. B., Bossone S. A. and Patel A. J. (1992). myc function and regulation. *Annu. Rev. Biochem.* 61, 809-858. 10.1146/annurev.bi.61.070192.004113 1497324

[DEV190231C41] Meyer N. and Penn L. Z. (2008). Reflecting on 25 years with MYC. *Nat. Rev. Cancer* 8, 976-990. 10.1038/nrc2231 19029958

[DEV190231C42] Mitchell N. C., Johanson T. M., Cranna N. J., Er A. L. J., Richardson H. E., Hannan R. D. and Quinn L. M. (2010). Hfp inhibits Drosophila myc transcription and cell growth in a TFIIH/Hay-dependent manner. *Development (Cambridge, England)* 137, 2875-2884. 10.1242/dev.049585 20667914

[DEV190231C43] Mitchell N. C., Tchoubrieva E. B., Chahal A., Woods S., Lee A., Lin J. I., Parsons L., Jastrzebski K., Poortinga G., Hannan K. M. et al. (2015). S6 Kinase is essential for MYC-dependent rDNA transcription in Drosophila. *Cell. Signal.* 27, 2045-2053. 10.1016/j.cellsig.2015.07.018 26215099

[DEV190231C44] Moshkovich N., Nisha P., Boyle P. J., Thompson B. A., Dale R. K. and Lei E. P. (2011). RNAi-independent role for Argonaute2 in CTCF/CP190 chromatin insulator function. *Genes Dev.* 25, 1686-1701. 10.1101/gad.16651211 21852534 PMC3165934

[DEV190231C45] Okamura K., Ishizuka A., Siomi H. and Siomi M. C. (2004). Distinct roles for Argonaute proteins in small RNA-directed RNA cleavage pathways. *Genes Dev.* 18, 1655-1666. 10.1101/gad.1210204 15231716 PMC478188

[DEV190231C46] Oskarsson T. and Trumpp A. (2005). The Myc trilogy: lord of RNA polymerases. *Nat. Cell Biol.* 7, 215-217. 10.1038/ncb0305-215 15738972

[DEV190231C47] Parisi C., Giorgi C., Batassa E. M., Braccini L., Maresca G., D'agnano I., Caputo V., Salvatore A., Pietrolati F., Cogoni C. et al. (2011). Ago1 and Ago2 differentially affect cell proliferation, motility and apoptosis when overexpressed in SH-SY5Y neuroblastoma cells. *FEBS Lett.* 585, 2965-2971. 10.1016/j.febslet.2011.08.003 21846468

[DEV190231C48] Peter A., Schöttler P., Werner M., Beinert N., Dowe G., Burkert P., Mourkioti F., Dentzer L., He Y., Deak P. et al. (2002). Mapping and identification of essential gene functions on the X chromosome of Drosophila. *EMBO Rep.* 3, 34-38. 10.1093/embo-reports/kvf012 11751581 PMC1083931

[DEV190231C49] Pirrotta V. and Li H.-B. (2012). A view of nuclear Polycomb bodies. *Curr. Opin. Genet. Dev.* 22, 101-109. 10.1016/j.gde.2011.11.004 22178420 PMC3329586

[DEV190231C50] Poortinga G., Wall M., Sanij E., Siwicki K., Ellul J., Brown D., Holloway T. P., Hannan R. D. and McArthur G. A. (2011). c-MYC coordinately regulates ribosomal gene chromatin remodeling and Pol I availability during granulocyte differentiation. *Nucleic Acids Res.* 39, 3267-3281. 10.1093/nar/gkq1205 21177653 PMC3082905

[DEV190231C51] Poortinga G., Quinn L. M. and Hannan R. D. (2014). Targeting RNA polymerase I to treat MYC-driven cancer. *Oncogene* 34, 403-412. 10.1038/onc.2014.13 24608428

[DEV190231C52] Pushpavalli S. N. C. V. L., Bag I., Pal-Bhadra M. and Bhadra U. (2012). Drosophila Argonaute-1 is critical for transcriptional cosuppression and heterochromatin formation. *Chromosome Res.* 20, 333-351. 10.1007/s10577-012-9279-y 22476395 PMC3323821

[DEV190231C53] Schuijers J., Manteiga J. C., Weintraub A. S., Day D. S., Zamudio A. V., Hnisz D., Lee T. I. and Young R. A. (2018). Transcriptional dysregulation of MYC reveals common enhancer-docking mechanism. *Cell Rep.* 23, 349-360. 10.1016/j.celrep.2018.03.056 29641996 PMC5929158

[DEV190231C54] Shevtsov S. P. and Dundr M. (2011). Nucleation of nuclear bodies by RNA. *Nat. Cell Biol.* 13, 167-173. 10.1038/ncb2157 21240286

[DEV190231C55] Shimada Y., Mohn F. and Bühler M. (2016). The RNA-induced transcriptional silencing complex targets chromatin exclusively via interacting with nascent transcripts. *Genes Dev.* 30, 2571-2580. 10.1101/gad.292599.116 27941123 PMC5204350

[DEV190231C56] Shiue C.-N., Berkson R. G. and Wright A. P. H. (2009). c-Myc induces changes in higher order rDNA structure on stimulation of quiescent cells. *Oncogene* 28, 1833-1842. 10.1038/onc.2009.21 19270725

[DEV190231C57] Taub R., Kirsch I., Morton C., Lenoir G., Swan D., Tronick S., Aaronson S. and Leder P. (1982). Translocation of the c-myc gene into the immunoglobulin heavy chain locus in human Burkitt lymphoma and murine plasmacytoma cells. *Proc. Natl. Acad. Sci. USA* 79, 7837-7841. 10.1073/pnas.79.24.7837 6818551 PMC347444

[DEV190231C58] Thomson D. W., Pillman K. A., Anderson M. L., Lawrence D. M., Toubia J., Goodall G. J. and Bracken C. P. (2015). Assessing the gene regulatory properties of Argonaute-bound small RNAs of diverse genomic origin. *Nucleic Acids Res.* 43, 470-481. 10.1093/nar/gku1242 25452337 PMC4288155

[DEV190231C59] Vennstrom B., Sheiness D., Zabielski J. and Bishop J. M. (1982). Isolation and characterization of c-myc, a cellular homolog of the oncogene (v-myc) of avian myelocytomatosis virus strain 29. *J. Virol.* 42, 773-779. 10.1128/JVI.42.3.773-779.1982 6284994 PMC256910

[DEV190231C60] Wang Q., Taliaferro J. M., Klibaite U., Hilgers V., Shaevitz J. W. and Rio D. C. (2016). The PSI–U1 snRNP interaction regulates male mating behavior in Drosophila. *Proc. Natl. Acad. Sci. USA* 113, 5269-5274. 10.1073/pnas.1600936113 27114556 PMC4868454

[DEV190231C61] Woolnough J. L., Atwood B. L. and Giles K. E. (2015). Argonaute 2 binds directly to tRNA genes and promotes gene repression in cis. *Mol. Cell. Biol.* 35, 2278-2294. 10.1128/MCB.00076-15 25918241 PMC4456445

[DEV190231C62] Zaytseva O. and Quinn L. M. (2017). Controlling the master: chromatin dynamics at the MYC promoter integrate developmental signaling. *Genes (Basel)* 8, 118 10.3390/genes8040118 PMC5406865 28398229

[DEV190231C63] Zhang J. and Chen Q. M. (2013). Far upstream element binding protein 1: a commander of transcription, translation and beyond. *Oncogene* 32, 2907-2916. 10.1038/onc.2012.350 22926519 PMC4968413

