# Supplementary material for: Transcriptional repression of Myc underlies the tumour suppressor function of AGO1 in Drosophila
Source: Development. 2020 Jun 11;147(11):dev190231. doi: 10.1242/dev.190231 (PMC7295588; doi:10.1242/dev.190231)
Supplement: Supplementary information [file develop-147-190231-s1.pdf]

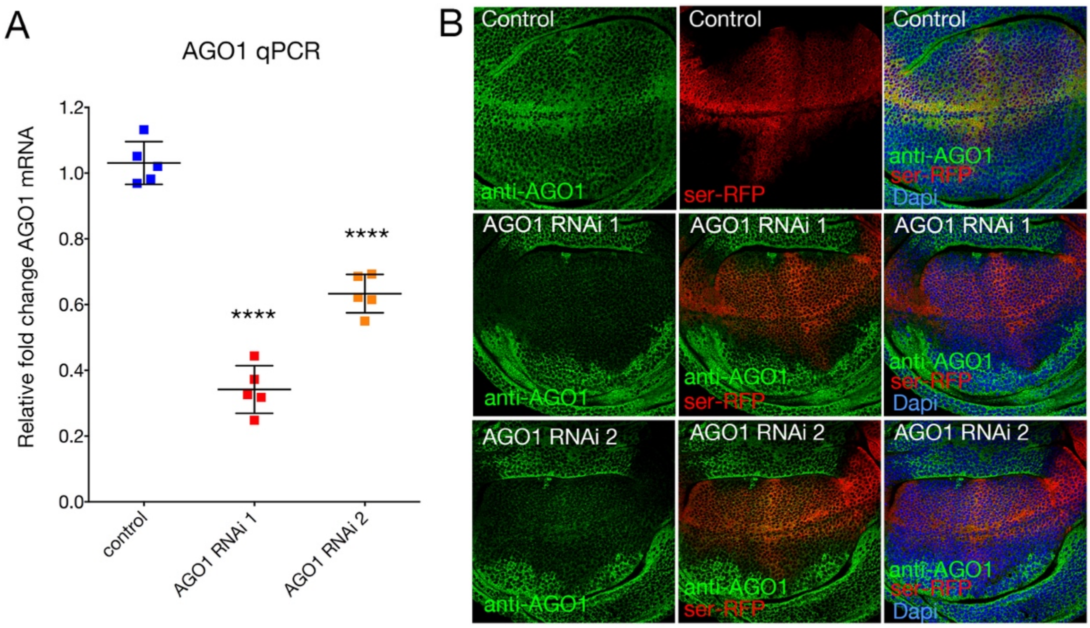

Fig. S1. *AGO1* RNAi lines decrease AGO1 mRNA and protein. (A) qPCR for control, *UAS-AGO1* RNAi 1, *UAS-AGO1* RNAi 2 on 3rd instar larval wing discs. **(B)** Wing imaginal discs with *ser*-GAL4 driven *AGO1* RNAi in *UAS*-RFP-marked cells stained with AGO1 antibody (green) and DNA stain (blue). Anti-AGO1 staining was performed in the background of *tub*-GAL80ts and conducted 24 h after shift to 29°C.

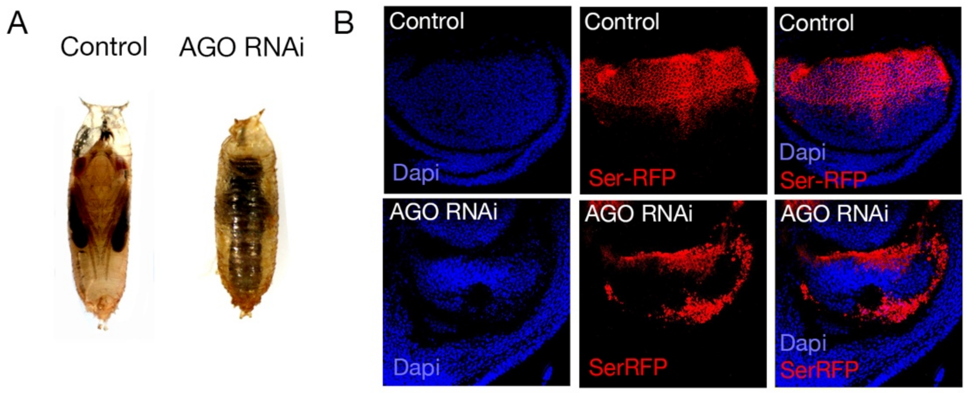

Fig. S2. *AGO1* RNAi drives cell death and pupal lethality (A) *serrate*-GAL4 driven *AGO1* RNAi resulted in pupal lethality and (B) cell death in third instar wing discs. DNA in blue and RFP in red.

Development • Supplementary information

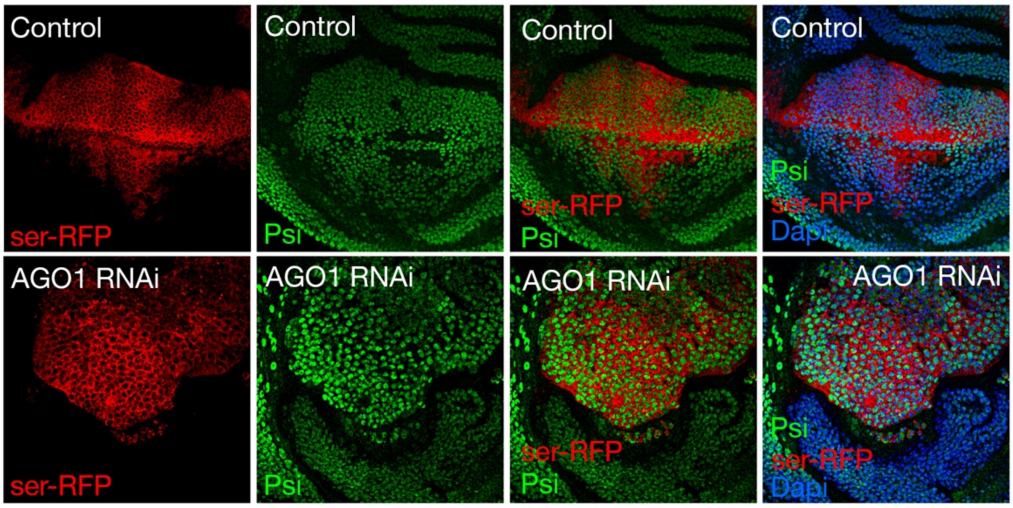

Fig. S3. *AGO1* RNAi increases Psi protein levels. *serrate*-GAL4 driven *AGO1* RNAi compared with control. Anti-Psi antibody (green), RFP (red) DNA (blue).

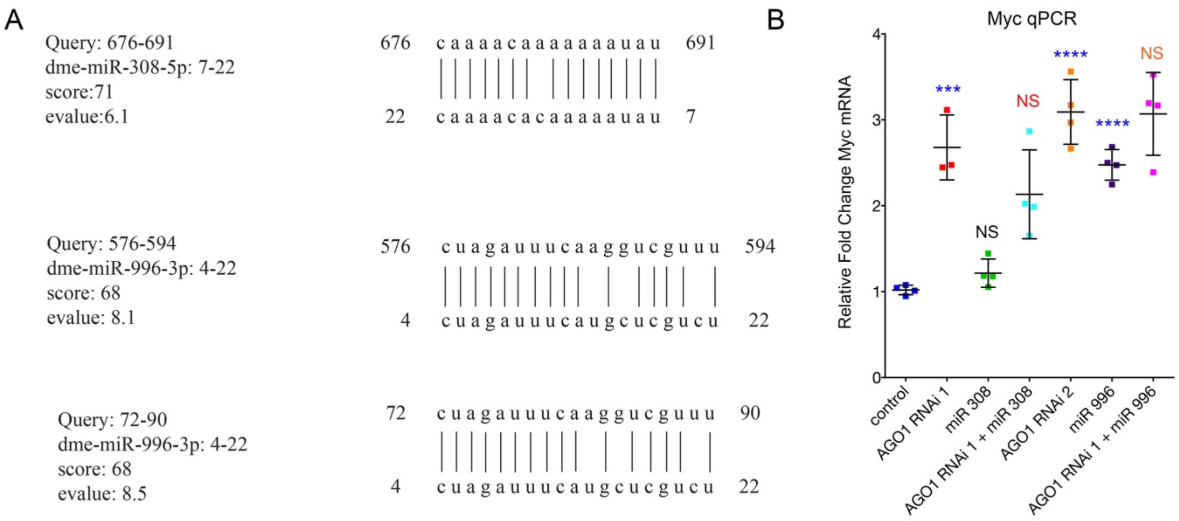

Fig. S4. Neither miR-996 nor miR-308 drive *Myc* mRNA turnover in the wing. (A) Seed sequence for miR-308 and miR-996 and base pairing with *Myc*. (B) AGO1 and *Myc* qPCR in larval wing discs for *AGO1* RNAi, miR-308, miR-996, and for *AGO1* RNAi in combination with either miR-308 or 996.

Development • Supplementary information

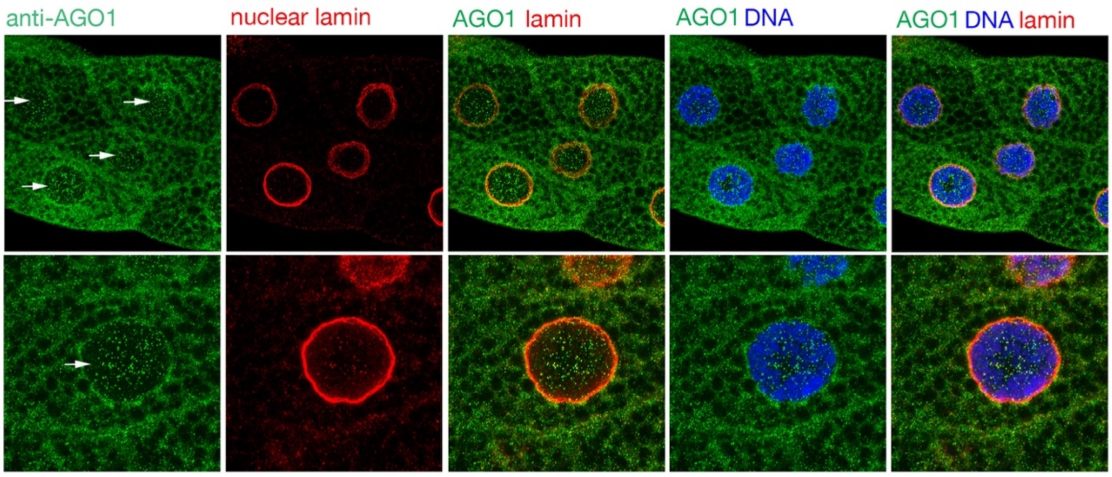

Fig. S5. Wild type third instar salivary glands stained with AGO1 antibody (green), nuclear lamin (red) and DNA (blue).

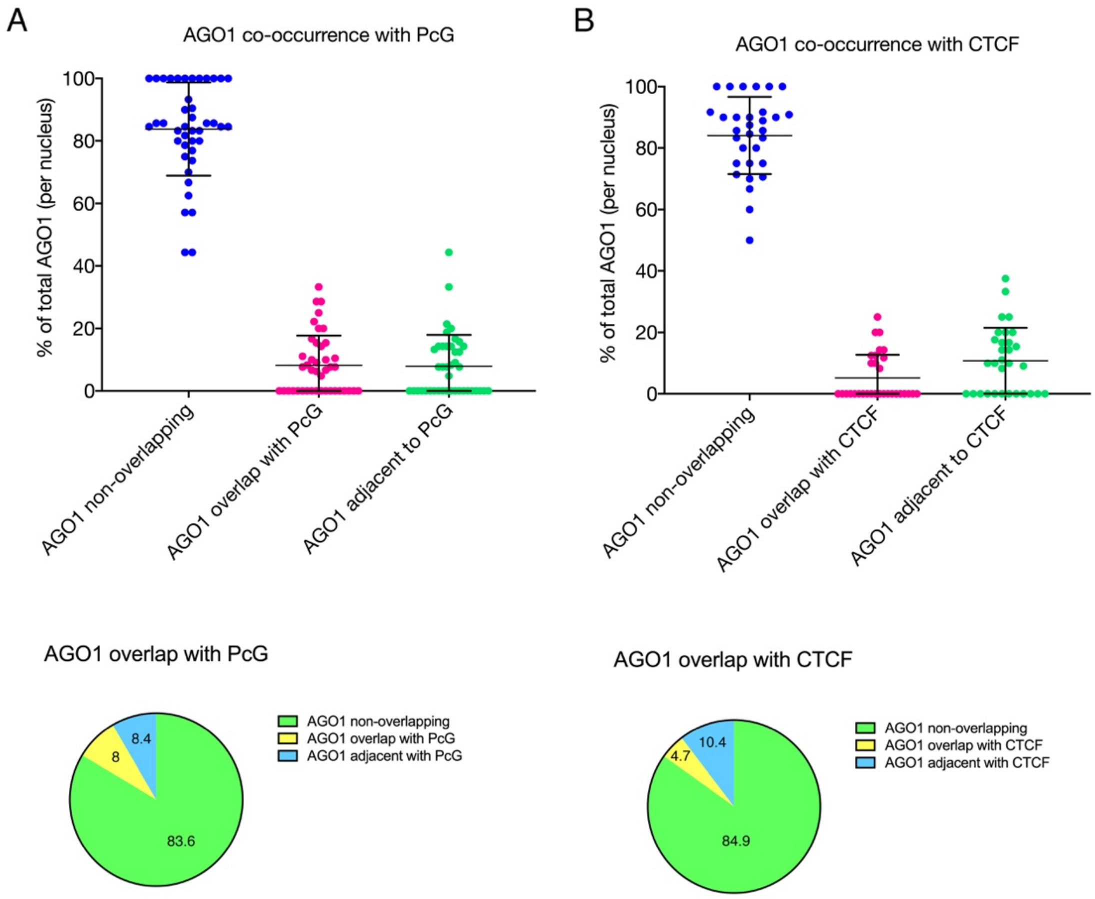

Fig. S6. Quantification of co-occurrence between AGO1 and (A) PcG or (B) CTCF bodies, expressed as percentage of total AGO1 puncta per nucleus.

Development • Supplementary information
