# Supplementary material for: Transcriptional repression of Myc underlies the tumour suppressor function of AGO1 in Drosophila
Source: Development. 2020 Jun 11;147(11):dev190231. doi: 10.1242/dev.190231 (PMC7295588; doi:10.1242/dev.190231)
Supplement: Reviewer comments [file develop-147-190231_review_history.pdf]

Transcriptional repression of Myc underlies the tumour suppressor function of AGO1 in *Drosophila*

Olga Zaytseva, Naomi C. Mitchell, Linna Guo, Owen J. Marshall, Linda M. Parsons, Ross D. Hannan, David L. Levens and Leonie M. Quinn
DOI: 10.1242/dev.190231

Editor: Haruhiko Koseki

Review timeline
Original submission: 9 March 2020
Editorial decision: 31 March 2020
First revision received: 15 April 2020
Accepted: 27 April 2020

Original submission

First decision letter

MS ID#: DEVELOP/2020/190231

MS TITLE: Transcriptional repression of Myc underlies AGO1's tumour suppressor function

AUTHORS: Olga Zaytseva, Naomi C Mitchell, Linna Guo, Owen Marshall, Linda M Parsons, Ross D Hannan, David L Levens, and Leonie M Quinn

I have now received all the referees reports on the above manuscript, and have reached a decision. The referees' comments are appended below, or you can access them online: please go to BenchPress and click on the 'Manuscripts with Decisions' queue in the Author Area.

The overall evaluation is positive and we would like to publish a revised manuscript in Development, provided that the referees' comments can be satisfactorily addressed. Please attend to all of the reviewers' comments in your revised manuscript and detail them in your point-by-point response. If you do not agree with any of their criticisms or suggestions explain clearly why this is so.

We are aware that you may currently be unable to access the lab to undertake experimental revisions. If it would be helpful, we encourage you to contact us to discuss your revision in greater detail. Please send us a point-by-point response indicating where you are able to address concerns raised (either experimentally or by changes to the text) and where you will not be able to do so within the normal timeframe of a revision. We will then provide further guidance. Please also note that we are happy to extend revision timeframes as necessary.

Reviewer 1

Advance summary and potential significance to field

The observation that AGO1 plays a direct role in the transcriptional regulation of myc is a significant advance for our understanding of how growth control is regulated during development. It is also notable for its description a transcriptional role for AGO1. Combined with previous data

© 2020. Published by The Company of Biologists under the terms of the Creative Commons Attribution License (https://creativecommons.org/licenses/by/4.0/).

showing that AGO1 can affect myc mRNA stability and translation of the mRNA, these new data firmly cement AGO1 as a critical regulation of myc levels through multiple mechanisms.

Comments for the author

The manuscript by Zaytseva shows a new role for AGO1 in the transcriptional regulation of myc. Combined with previous data showing that AGO1 can affect myc mRNA stability and translation of the mRNA, these new data firmly cement AGO1 as a critical regulation of myc levels through multiple mechanisms. The data shown are clear and robust, and support the conclusions made by the authors. This is an interesting and important piece of work for which I have only minor concerns.

Experimental questions:
In figure 2, the suppression of AGO1 haplo-insufficiency by Psi1 knockdown is somewhat complicated by the fact that both genotypes show a phenotype by themselves – AGO1 +/- animals have larger than wild-type wings and Psi knockdown causes smaller wings. The combination of the 2 is an intermediate effect. Is it possible to supplement these analyses by testing whether AGO1+/- is suppressed by Psi +/- (which I assume does not have a haplo phenotype)?

In Figure 6, the authors show limited co-localization between Pc or CTCF and AGO1. The extent to which either of these factors could contribute to the growth (myc) effects caused by reduced AGO1 is not clear.
Would it be possible to combine reduced (heterozygote or RNAi) Pc or CTCF with AGO1i (e.g. any of the assays presented in Figure 1, 2, 3, 4 or 5). This seems particularly relevant for Pc in light of a publication from Goofliffe et al. 2006 (Genes Dev), who showed that Pc was required for Myc autoregulation (autorepression).
Is it possible that the AGO1 phenotypes are caused by a failure of Myc to autoregulate?

Text/Figure clarifications:
Figures 1 and 7 show “mock” IPs for co-IP and ChIP, respectively. I could not find what exactly this meant. Is it an IgG control?

In Figure 3, gene naming is not consistent between the graph in C and the text (e.g. RPs19 vs RpS19).

In Figure 4, clarification of statistical indicators (e.g. brackets) would make this graph much clearer. I assume that AGO1i+Psii is statistically different from AGO alone? Is Myc RNAi (alone) being compared to control? Is AGO1i+Myci different to Myci alone AND AGO1i alone, or only one of these?

The conclusion from Figure 4 was that “the increased ribosome biogenesis and cell overgrowth associated with AGO1 depletion is dependent on the Psi-Myc axis”. While I agree that both Psi and Myc independently suppress the AGO1 RNAi phenotype, is that sufficient to conclude that it’s the Psi-Myc axis? Are Psi and Myc necessarily working together? Perhaps it’s not clear to me what “axis” means.

While the authors state that Psi has essential roles in myc transcriptional control, myc mRNA levels seem unaffected by Psi knockdown in Figure 5A. The discussion refers to this as a “modest decrease” in myc mRNA. A little more explanation (and clear indication as to whether any decrease is statistically significant) would be nice. In addition, there is no significance indicator for myc RNAi alone. This is important information to be able to interpret the data. Is Myci+AGO1i different to Myci alone or to AGO1i alone?

It was not clear why some graphs had colored significance indicators (**** etc) and some were simply black.

The sentence in the 1st paragraph is missing word(s) or something “The MYC transcription factor and growth regulator has been studied extensively since identification as an oncogene in the early eighties (Vennstrom et al., 1982), and given MYC overexpression due to chromosomal translocation

© 2020. Published by The Company of Biologists under the terms of the Creative Commons Attribution License (https://creativecommons.org/licenses/by/4.0/).

directly drives malignant transformation in Burkitt's Lymphoma (Dalla-Favera et al., 1982; Taub et al., 1982).

The statement that "AGO1 behaves as a tumor suppressor during Drosophila development" in the discussion is probably a bit bold. It certainly shows activities in Drosophila that are consistent with it having tumor suppressive capabilities in human cells.

Reviewer 2

Advance summary and potential significance to field

In this report, Zaytseva et al show, using Drosophila imaginal tissues, that Ago functions as a tumour suppressor by inhibiting Myc and Myc-dependent ribosome biogenesis. They show that these effects are due to Ago functioning at the Myc promoter to regulate Psi and Pol II transcription of Myc

Overall this is a very good paper. The data are well presented (the use of dot plots is excellent and an example to all) and the conclusions and inferences are sound. Given that the key regulators investigates here (Ago, Psi, Myc) are all conserved, the key findings of the paper will be relevant for our understanding of tissue growth during development in other animals.

Comments for the author

I support publication of this paper - all my comments below shouldn't require any more experimental work.

Comments

1. Fig 2b,c – authors report that Ago heterozygosity leads to increased wing size, and hence conclude that Ago1 functions as a negative regulator of growth (i.e. a tumour suppressor). This conclusion would be strengthened if the authors have any data showing that these effects are cell-autonomous. For example, it would be good to rule out the possibility that Ago heterozygosity doesn't function in some non-autonomous way to lead to a delay in larval development, and hence more time for imaginal tissues to grow and give rise to larger organs. Does Ago RNAi in imaginal tissues (as used in expts in the other Figs) lead to increase cell and tissue size? (from the images it looks like the cells are bigger – can this be quantified from the available data - e.g. nuclear density per fixed area? cell volume (if outlines are marked)?
2. In Fig 5C, the increase in Myc protein levels in the Ago1 RNAi clones was not so clear. Would it be possible for the authors to quantify Myc protein staining in their available clonal Ago1 RNAi/Myc immunostaining images?
3. Minor point - I was a little unclear about how the experiment in Fig 7C was performed – the authors say they incubated 'heads' in amanatin – does this mean all the anterior region (brain, discs etc.)? – I imagine the goal here was to as quickly as possible isolate and incubate imaginal tissues (because that's where the GAL4 driver expresses transgenes?) in drug and then freeze them for the qPCR. It would be worth explaining what driver was used in these expts. Also, in the course of the subsequent qPCRs expts, did the authors test expression of any genes to indicate that they had equal amounts of imaginal tissues (or Ser-GAL4 expressing tissues) in their samples?

First revision

Author response to reviewers' comments

We thank both reviewers for their positive feedback and suggestions, which have significantly improved the manuscript. Fortunately, given the COVID-19 pandemic has forced our University into shutdown (25th of March-26th of June), we have been able to address all comments from the

© 2020. Published by The Company of Biologists under the terms of the Creative Commons Attribution License (https://creativecommons.org/licenses/by/4.0/).

reviewers without the requirement for further experiments. Detailed responses for each of the comments from Reviewer 1 and 2 are listed below. All changes to the manuscript have been incorporated as outlined and highlighted in red text of the manuscript document for clarity.

Reviewer 1 Comments for the author
Reviewer 1.1 The manuscript by Zaytseva shows a new role for AGO1 in the transcriptional regulation of myc. Combined with previous data showing that AGO1 can affect myc mRNA stability and translation of the mRNA, these new data firmly cement AGO1 as a critical regulation of myc levels through multiple mechanisms. The data shown are clear and robust, and support the conclusions made by the authors. This is an interesting and important piece of work for which I have only minor concerns.

Experimental questions:
Reviewer 1.2 In figure 2, the suppression of AGO1 haplo-insufficiency by Psi1 knockdown is somewhat complicated by the fact that both genotypes show a phenotype by themselves – AGO1 +/- animals have larger than wild-type wings and Psi knockdown causes smaller wings. The combination of the 2 is an intermediate effect. Is it possible to supplement these analyses by testing whether AGO1+/- is suppressed by Psi +/- (which I assume does not have a haplo phenotype)?

Response 1.2 - The reviewer is correct. The experiment in Figure 2 could potentially be confounded by additive effects due to the impaired growth from Psi knockdown and the overgrowth in the AGO1 +/- background. Indeed, our genome wide binding studies (in preparation) show Psi regulates growth by targeting multiple independent pathways, which could contribute to additive affects. Nonetheless, our larval stage analysis (Figure 4) shows that although Psi depletion alone does not alter nucleolar size in the larval stage, it does suppress the expansion of the nucleolus caused by AGO1 knockdown. Thus, Psi knockdown does suppress nucleolar expansion due to AGO1 depletion, suggesting Psi is required for the overgrowth. These points have been clarified in the text.

Discussion added in context of Figure 2:
"However, we need to be somewhat cautious in our interpretation of these data as both genotypes result in a phenotype alone, where the outcome may be an intermediate phenotype between the larger wings from AGO1 heterozygotes and impaired growth from Psi knockdown."

Discussion in context of Figure 4:
"Myc depletion reduced nucleolar expansion in AGO1 knockdown wing cells down to the control range (Fig.4A,B). Co-depletion of the Myc-regulator Psi also significantly decreased nucleolar size compared with AGO1 knockdown alone (Fig. 4A,B). Importantly, given the intermediate effect between ser-GAL4 driven Psi knockdown and AGO1 heterozygotes in the adult wing (Fig. 2), Psi depletion alone did not modify nucleolar size."

Reviewer 1.3 In Figure 6, the authors show limited co-localization between Pc or CTCF and AGO1. The extent to which either of these factors could contribute to the growth (myc) effects caused by reduced AGO1 is not clear. Would it be possible to combine reduced (heterozygote or RNAi) Pc or CTCF with AGO1i (e.g. any of the assays presented in Figure 1, 2, 3, 4 or 5). This seems particularly relevant for Pc in light of a publication from Goofliffe et al. 2005 (Genes Dev), who showed that Pc was required for Myc autoregulation (autorepression). Is it possible that the AGO1 phenotypes are caused by a failure of Myc to autoregulate?

Response 1.3 - The Goodliffe et al 2005 paper showing Pc is required for Myc autorepression in the Drosophila embryo, together with the partial overlap between AGO1 and PcG or CTCF, might suggest Pc could mediate AGO1-dependent transcriptional control. However, the AGO1 phenotypes we observe are unlikely to be a consequence of failure in autoregulation. The approach used in the autoregulation studies involves investigation of consequences of Myc overexpression from an exogenous promoter and are, thus, not immediately applicable to our analysis of endogenous regulation of Myc transcription. Rather, our data suggest that AGO1 is required for repression of endogenous Myc transcription, binding the Myc promoter under normal conditions and depletion itself being sufficient to increase Myc transcription. The Myc autoregulation observed by Goodliffe et al. is a consequence of a massive increase in Myc, in the range of 100x over endogenous levels of Myc in the embryo in stark contrast to the 3-5 fold increase associated with AGO1 knockdown in the wing (Figure 5A). In a similar vein, super-enhancers control MYC transcription via CTCF in the

© 2020. Published by The Company of Biologists under the terms of the Creative Commons Attribution License (https://creativecommons.org/licenses/by/4.0/).

context of high-MYC cancers, and although it will be of interest to determine whether such non-physiological levels of MYC expression are mediated by AGO proteins, this would constitute an extensive stand-alone study beyond the scope of this manuscript.

Thus, we have highlighted the potential significance of the overlap between AGO1 and Pc/CTCF, including an integration of the findings from Goodliffe et. al. 2005, into the discussion as follows:

Myc transcriptional autorepression, modelled in the Drosophila embryo via overexpression of Myc from an exogenous promoter, leads to repression of the endogenous Myc locus in a Pc-dependent manner (Goodliffe et al., 2005). This, together with the partial overlap between AGO1 and PcG in wing imaginal disc cells (Figure 6C), might suggest Pc mediates transcriptional autorepression of Myc via AGO1. In contrast to our studies, where AGO1 depletion phenotypes are associated with a moderate (>3- to 5-fold) increase in Myc, autoregulation in the embryo was investigated in response to non-physiological increases in Myc (>100x over endogenous levels) (Goodliffe et al., 2005). Thus, our data suggest AGO1 binds the Myc promoter under normal conditions and is required for repression of endogenous Myc transcription (Figure 5A), but whether AGO1 is required for Pc-dependent Myc-autorepression requires further investigation. In a similar vein, super-enhancers control human MYC transcription via CTCF in the context of high-MYC cancers (Schuijers et al., 2018). Thus, failed Pc-dependent autorepression and/or defective repression of super-enhancers via CTCF could further elevate MYC to promote cancer progression. Given the observed overlap between AGO1 and Pc/CTCF in the Drosophila wing, future studies determining whether AGO1 interacts with Pc and/or CTCF to control autoregulatory feedback on Myc transcription in the context of tumorigenesis will be of great interest.

Text/Figure clarifications:
Reviewer 1.4 - Figures 1 and 7 show “mock” IPs for co-IP and ChIP, respectively. I could not find what exactly this meant. Is it an IgG control?

Response 1.4 - “Mock IP” refers to control performed without the use of antibody, not IgG control. This has been clarified in the Methods section and figure legends.

Reviewer 1.5 - In Figure 3, gene naming is not consistent between the graph in C and the text (e.g. RPs19 vs RpS19).

Response 1.5 - The labelling on the figure has been amended to be consistent with the text.

Reviewer 1.6 - In Figure 4, clarification of statistical indicators (e.g. brackets) would make this graph much clearer. I assume that AGO1i+Psii is statistically different from AGO alone? Is Myc RNAi (alone) being compared to control? Is AGO1i+Myci different to Myci alone AND AGO1i alone, or only one of these?

Response 1.6 - Consistent with the other figures, the indicators are coloured according to the group to which the comparison is made. Each RNAi alone are compared to control, indicated by blue stars (in the case of Psi RNAi the lack of statistical significance was added to the figure). To answer the particular question whether Psi or Myc RNAi modify AGO RNAi, each of the double knockdown conditions is compared to AGO RNAi alone (red stars).

Reviewer 1.7 - The conclusion from Figure 4 was that “the increased ribosome biogenesis and cell overgrowth associated with AGO1 depletion is dependent on the Psi-Myc axis”. While I agree that both Psi and Myc independently suppress the AGO1 RNAi phenotype, is that sufficient to conclude that it’s the Psi-Myc axis? Are Psi and Myc necessarily working together? Perhaps it’s not clear to me what “axis” means.

Response 1.7 - We agree this was not clear. We have now reworded to state “the increased ribosome biogenesis and cell overgrowth associated with AGO1 depletion is dependent on both Psi and Myc”.

Reviewer 1.8 - While the authors state that Psi has essential roles in myc transcriptional control, myc mRNA levels seem unaffected by Psi knockdown in Figure 5A. The discussion refers to this as a “modest decrease” in myc mRNA. A little more explanation (and clear indication as to whether any

© 2020. Published by The Company of Biologists under the terms of the Creative Commons Attribution License (https://creativecommons.org/licenses/by/4.0/).

decrease is statistically significant) would be nice. In addition, there is no significance indicator for myc RNAi alone. This is important information to be able to interpret the data. Is Myci+AGO1i different to Myci alone or to AGO1i alone?

Response 1.8 - We apologise as this was not clear, the "modest decrease" in Myc mRNA refers to the combined AGO1 RNAi/Psi RNAi compared to AGO1 RNAi alone. We have amended the text to clarify: "Psi co-knockdown only modestly decreased Myc mRNA levels in AGO1-depleted wings".

The significance indicator was added showing significant Myc depletion in Myc RNAi compared to control. The indicator for AGO1 RNAi/Myc RNAi was incorrect and has been replaced by a comparison to control to verify Myc knockdown in AGO1 RNAi background.

Reviewer 1.9 - It was not clear why some graphs had colored significance indicators (**** etc) and some were simply black.

Response 1.9 - The black colour of the indicators in Figure 3 was indeed inconsistent with the rest of the figures. The indicators in Figure 3 have been coloured to reflect comparison with the appropriate controls.

Reviewer 1.10 - The sentence in the 1st paragraph is missing word(s) or something "The MYC transcription factor and growth regulator has been studied extensively since identification as an oncogene in the early eighties (Vennstrom et al., 1982), and given MYC overexpression due to chromosomal translocation directly drives malignant transformation in Burkitt's Lymphoma (Dalla-Favera et al., 1982; Taub et al., 1982).

Response 1.10 - This sentence has now been reworded to read "The MYC transcription factor and growth regulator has been studied extensively since identification as an oncogene in the early eighties (Vennstrom et al., 1982) when MYC overexpression due to chromosomal translocation was found to drive malignant transformation in Burkitt's Lymphoma (Dalla-Favera et al., 1982; Taub et al., 1982).

Reviewer 1.11 - The statement that "AGO1 behaves as a tumor suppressor during Drosophila development" in the discussion is probably a bit bold. It certainly shows activities in Drosophila that are consistent with it having tumor suppressive capabilities in human cells.

Response 1.11 - We agree and have softened this to read "AGO1 behaves as a growth inhibitor during Drosophila development, … Consistent with AGO1 having tumour suppressor activity, across a wide range of human cancers…"

Reviewer 2 Comments for the author
I support publication of this paper - all my comments below shouldn't require any more experimental work.

Reviewer 2.1. Fig 2b,c – authors report that Ago heterozygosity leads to increased wing size, and hence conclude that Ago1 functions as a negative regulator of growth (i.e. a tumour suppressor). This conclusion would be strengthened if the authors have any data showing that these effects are cell-autonomous. For example, it would be good to rule out the possibility that Ago heterozygosity doesn't function in some non-autonomous way to lead to a delay in larval development, and hence more time for imaginal tissues to grow and give rise to larger organs. Does Ago RNAi in imaginal tissues (as used in expts in the other Figs) lead to increase cell and tissue size? (from the images it looks like the cells are bigger – can this be quantified from the available data - e.g. nuclear density per fixed area? cell volume (if outlines are marked)?

Response 2.1. This is a good point as non-autonomous effects can result in tissue overgrowth as a consequence of developmental timing delays. However, the lack of an extended time to eclosure for the AGO1 heterozygotes suggests this is unlikely to contribute to the wing overgrowth. The increase in the nucleolar compartment in the AGO1 knockdown cells in the wing (Figure 3) also suggests AGO1 functions cell autonomously to increase ribosome biogenesis i.e. cell growth. Nevertheless, since nucleolar size is an indirect measure of growth, we have also quantified cell area using the membrane-bound CD8-RFP signal in control and AGO1-depleted imaginal wing disc

© 2020. Published by The Company of Biologists under the terms of the Creative Commons Attribution License (https://creativecommons.org/licenses/by/4.0/).

cells. We have now added data to show AGO1 knockdown increased cell area with CD8-RFP (Figure 3C).

Reviewer 2.2. In Fig 5C, the increase in Myc protein levels in the Ago1 RNAi clones was not so clear. Would it be possible for the authors to quantify Myc protein staining in their available clonal Ago1 RNAi/Myc immunostaining images?

Response 2.2. The reviewer makes a good suggestion to quantify Myc protein levels, which is best performed as a ratio of the intensity of Myc antibody staining in RFP-positive cells of the dorsal compartment, compared to RFP-negative cells of the ventral compartment. Quantification of Myc, which is expressed at similar levels in both compartments, results in a ratio of around 1:1 in the control wing. We did attempt to quantify Myc intensity but were unable to do so robustly because AGO1 knockdown in the dorsal compartment severely diminished the ventral compartment and prevented reliable normalisation. We predict the destruction of the ventral compartment may be due to confounding effects of cell competition due to the Myc-high, AGO1-depleted cells. Although we were unable to quantify the Myc protein abundance directly, we show a significantly increased expression of direct Myc targets, Polr1c and Cad, in AGO1-depleted tissues (presented in Figure 5D,E) consistent with increased Myc protein function.

Reviewer 2.3. Minor point - I was a little unclear about how the experiment in Fig 7C was performed – the authors say they incubated 'heads' in amanatin – does this mean all the anterior region (brain, discs etc.)? I imagine the goal here was to as quickly as possible isolate and incubate imaginal tissues (because that's where the GAL4 driver expresses transgenes?) in drug and then freeze them for the qPCR. It would be worth explaining what driver was used in these expts.

Response 2.3. Apologies this was unclear. The amanitin treatment was carried out on all anterior tissue including brains and imaginal discs to allow enough tissue to be collected rapidly. For expression in all head tissues we used the ubiquitous tubulin-GAL4 driver, temporally controlled using tub-GAL80ts. The latter was important to temporally limit AGO1 knockdown to 2 days i.e. prior to confounding effects of apoptosis. We have now clarified this in the methods.

Reviewer 2.4. Also, in the course of the subsequent qPCRs expts, did the authors test expression of any genes to indicate that they had equal amounts of imaginal tissues (or Ser-GAL4 expressing tissues) in their samples?

Response 2.4. All qPCR experiments were performed using the tubulin-GAL4 driver for expression of RNAi in all larval tissues. To normalise for any variability in the amount of tissue between samples, we have used cyp1 and tubulin as reference genes, which were selected for having high expression and little sample-to-sample variability as determined by RefFinder. The explanation of our normalisation strategy has been made clearer in the Methods section as follows: "Multiple candidate reference genes were analysed for stability in the AGO1 RNAi samples and control using RefFinder, and target gene expression was normalized to the mean of cyp1 and tubulin, selected for having high expression and smallest sample-to-sample variability".

Second decision letter

MS ID#: DEVELOP/2020/190231

MS TITLE: Transcriptional repression of Myc underlies AGO1's tumour suppressor function

AUTHORS: Olga Zaytseva, Naomi C Mitchell, Linna Guo, Owen Marshall, Linda M Parsons, Ross D Hannan, David L Levens, and Leonie M Quinn
ARTICLE TYPE: Research Article

I am happy to tell you that your manuscript has been accepted for publication in Development, pending our standard ethics checks.

© 2020. Published by The Company of Biologists under the terms of the Creative Commons Attribution License (https://creativecommons.org/licenses/by/4.0/).

Reviewer 1

Advance summary and potential significance to field

see previous review

Comments for the author

The authors have addressed all of my concerns.

Reviewer 2

Advance summary and potential significance to field

All the new additions to the paper are good.

Comments for the author

The revised manuscript addresses all my previous concerns. Congratulations on a very nice paper

© 2020. Published by The Company of Biologists under the terms of the Creative Commons Attribution License (https://creativecommons.org/licenses/by/4.0/).
